# Cryo-EM structures of human m^6^A writer complexes

**DOI:** 10.1038/s41422-022-00725-8

**Published:** 2022-09-27

**Authors:** Shichen Su, Shanshan Li, Ting Deng, Minsong Gao, Yue Yin, Baixing Wu, Chao Peng, Jianzhao Liu, Jinbiao Ma, Kaiming Zhang

**Affiliations:** 1 State Key Laboratory of Genetic Engineering, Collaborative Innovation Center of Genetics and Development, Multiscale Research Institute of Complex Systems, Department of Biochemistry and Biophysics, School of Life Sciences, Fudan University, Shanghai, China; 2 MOE Key Laboratory for Cellular Dynamics and Division of Life Sciences and Medicine, University of Science and Technology of China, Hefei, Anhui China; 3 MOE Key Laboratory of Macromolecular Synthesis and Functionalization, Department of Polymer Science and Engineering, Zhejiang University, Hangzhou, Zhejiang China; 4 National Facility for Protein Science in Shanghai, Zhangjiang Laboratory, Shanghai Advanced Research Institute, Chinese Academy of Science, Shanghai, China; 5 Guangdong Provincial Key Laboratory of Malignant Tumor Epigenetics and Gene Regulation, Guangdong-Hong Kong Joint Laboratory for RNA Medicine, RNA Biomedical Institute, Medical Research Center, Sun Yat-Sen Memorial Hospital, Sun Yat-Sen University, Guangzhou, Guangdong China

**Keywords:** Post-translational modifications, Cryoelectron microscopy

## Abstract

*N*^6^-methyladenosine (m^6^A) is the most abundant ribonucleotide modification among eukaryotic messenger RNAs. The m^6^A “writer” consists of the catalytic subunit m^6^A-METTL complex (MAC) and the regulatory subunit m^6^A-METTL-associated complex (MACOM), the latter being essential for enzymatic activity. Here, we report the cryo-electron microscopy (cryo-EM) structures of MACOM at a 3.0-Å resolution, uncovering that WTAP and VIRMA form the core structure of MACOM and that ZC3H13 stretches the conformation by binding VIRMA. Furthermore, the 4.4-Å resolution cryo-EM map of the MACOM–MAC complex, combined with crosslinking mass spectrometry and GST pull-down analysis, elucidates a plausible model of the m^6^A writer complex, in which MACOM binds to MAC mainly through WTAP and METTL3 interactions. In combination with in vitro RNA substrate binding and m^6^A methyltransferase activity assays, our results illustrate the molecular basis of how MACOM assembles and interacts with MAC to form an active m^6^A writer complex.

## Introduction

Over one hundred different RNA modifications have been identified in a variety of RNAs, including mRNA, rRNA, tRNA, snRNA, microRNA, and long noncoding RNA.^1^ Among these modifications, *N*^6^-methyladenosine (m^6^A) is the most prevalent and conserved ribonucleotide modification.^2^ m^6^A modification is enriched in coding sequences and 3′ untranslated regions (3′ UTRs) of eukaryotic mRNAs and plays crucial roles in numerous physiological and pathophysiological processes.^3,4^

Deposition of m^6^A onto RNAs is achieved by the m^6^A methyltransferase complex (m^6^A writer), a large holocomplex of ~1000 kDa in size that contains methyltransferase-like 3 and 14 (METTL3/14), Wilms’ tumor 1-associated protein (WTAP), KIAA1429 (VIRMA), Zinc finger CCCH domain-containing protein 13 (ZC3H13), RNA binding motif protein 15/15 paralog (RBM15/15B), and E3 ubiquitin ligase CBLL1 (HAKAI).^5–11^ The heterodimer of METTL3 and METTL14 forms the catalytic subunit of the m^6^A writer,^12^ also known as the m^6^A-METTL complex (MAC).^7^ Structural and biochemical studies have shown that METTL3 is the catalytic component that binds to the cofactor SAM, while METTL14 plays a key role in stabilizing the conformation of METTL3 for optimal substrate binding.^13–16^ In addition, WTAP, VIRMA, ZC3H13, HAKAI, and RBM15/RBM15B have been identified in various species to play regulatory roles in m^6^A writer activity and region-selective m^6^A methylation of mRNAs.^5–10^ These components form a regulatory subunit called the m^6^A-METTL-associated complex (MACOM)^7,17^ that is conserved in most eukaryotic species, such as flies, mice, humans, and plants. Protein–protein interaction analysis^18^ has revealed that the four components of MACOM, HAKAI, WTAP, VIRMA, and ZC3H13 can form a stable functional complex (hereinafter referred to as HWVZ) in human cells, which has been validated by co-fractionation experiments.^7,8,10^ WTAP was first identified as a METTL3-interacting protein in plants^19^ and was shown to be critical for the anchorage of the METTL3/14 heterodimer in nuclear speckles,^5^ probably through the interactions with Leader Helix (LH) of METTL3.^20^ VIRMA has been shown to be closely associated with WTAP and HAKAI in plants^21^ and to mediate preferential m^6^A mRNA methylation in the 3′ UTR.^10^ ZC3H13 is required for the nuclear localization of MACOM in mouse embryonic stem cells (mESCs)^8^ and for bridging WTAP–RBM15 interactions in flies.^7^ HAKAI interacts with other components in MACOM via its RING domain,^17,22^ which is necessary for the stability of MACOM components^22^ and m^6^A methylation in plants and flies.^21,23^ However, due to the lack of structural information of MACOM, how the components of MACOM are assembled and interact with MAC is not well understood at the molecular level.

Here, we use cryo-electron microscopy (cryo-EM) to resolve the structures of the complexes formed by human MACOM core components (HAKAI, WTAP, and VIRMA with or without ZC3H13) at 3.0-Å resolution, and the structure of the MACOM complexed with MAC at an overall resolution of 4.4 Å (the resolution of MAC region is relatively lower due to its flexibility). These three-dimensional (3D) structural information, in combination with crosslinking mass spectrometry and GST pull-down analysis, enable us to propose a plausible model for MACOM and MAC assembly to form an active m^6^A writer complex for RNA substrate binding and modification. This model facilitates understanding of the molecular function of MACOM and drug development for m^6^A-related diseases such as human cancers.^24^

## Results

### MACOM directly binds to RNA substrates and is essential for m^6^A writer activity

**Fig. 1:**
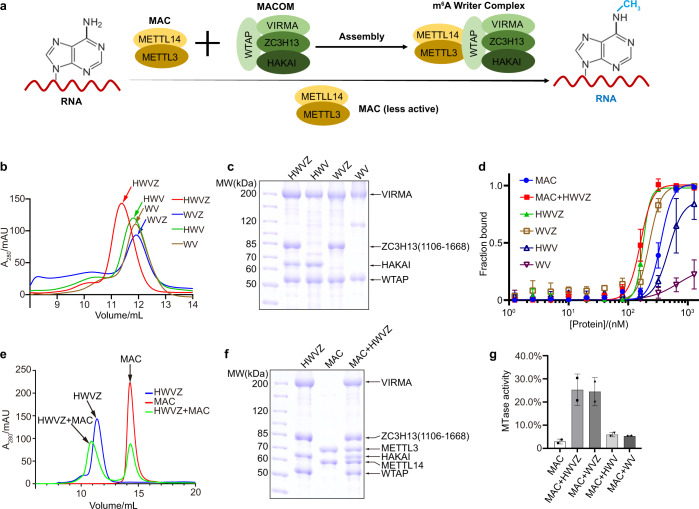
MACOM directly binds RNA substrates and is essential for m^6^A writer activity. **a** Model of RNA m^6^A methylation by MAC and MACOM. **b** Overlay view of size exclusion chromatography results of HWVZ, WVZ, HWV, and WV complexes. **c** SDS-PAGE of HWVZ, WVZ, HWV, and WV complexes, corresponding to **b**. **d** Binding affinities of the MAC, MAC + HWVZ, HWVZ, WVZ, HWV, and WV complexes with FAM-labeled *ACTB-1* RNA from EMSA experiments. Binding curves were generated using the means of three independent experiments, with standard deviation (SD) values indicated by error bars (*n* = 3). **e** Overlay view of size exclusion chromatography results of MAC, HWVZ, and MAC-HWVZ complexes. **f** SDS-PAGE of MAC, HWVZ, and MAC-HWVZ complexes, corresponding to **e**. **g** *ACTB-1* RNA *N*^6^-adenosine methylation activity of MAC with or without HWVZ, WVZ, HWV, and WV complex. Data are shown as the means ± SD (*n* = 2).

Previous studies have shown that each component of MACOM is essential for m^6^A modification in various species in vivo,^19,21–23^ and the catalytic subunit MAC is much less active when MACOM components are deleted (Fig. 1a). However, little is known about the properties of these components in vitro. After extensive trials of expression and purification, we obtained four complexes containing different MACOM components: HWVZ (containing HAKAI, WTAP, VIRMA, and ZC3H13 (1106–1668)), HWV (containing HAKAI, WTAP, and VIRMA), WVZ (containing WTAP, VIRMA, and ZC3H13 (1106–1668)), and WV (containing WTAP and VIRMA) (Fig. 1b, c). These four complexes possess different RNA substrate binding abilities according to electrophoretic mobility shift assay (EMSA) (Fig. 1d). HWVZ has the highest binding affinity (*K*_d_ = 214.0 ± 2.6 nM), and removing HAKAI weakly attenuates the binding affinity of WVZ (*K*_d_ = 256.3 ± 27.9 nM). In contrast, removing ZC3H13 has a dramatic effect, which reduces the binding affinities of HWV and WV complexes to *K*_d_ = 562.7 ± 76.6 nM and 826.3 ± 336.0 nM, respectively. Therefore, all components of MACOM contribute to RNA substrate binding, especially for the ZC3H13 subunit. Then, we reconstituted the m^6^A writer complex by assembling MACOM components with MAC. The m^6^A writer complex reconstituted by HWVZ and MAC exhibits much higher enzymatic activity than MAC alone (Fig. 1e–g). Removing HAKAI from the m^6^A writer complex has little effect on the activity (“MAC + WVZ” in Fig. 1g). However, removing ZC3H13 from the m^6^A writer complex dramatically reduces the enzymatic activity (“MAC + HWV” and “MAC + WV” in Fig. 1g). These results demonstrate that MACOM is essential for m^6^A writer activity and that ZC3H13 is a key component in MACOM. Notably, the RNA-binding ability of MAC is significantly weaker than the MACOM complex (Fig. 1d), suggesting that the lower enzymatic activity of MAC alone may be due to less RNA binding or the lack of active configuration in the absence of the other components.

### Overall structure of the human MACOM

**Fig. 2:**
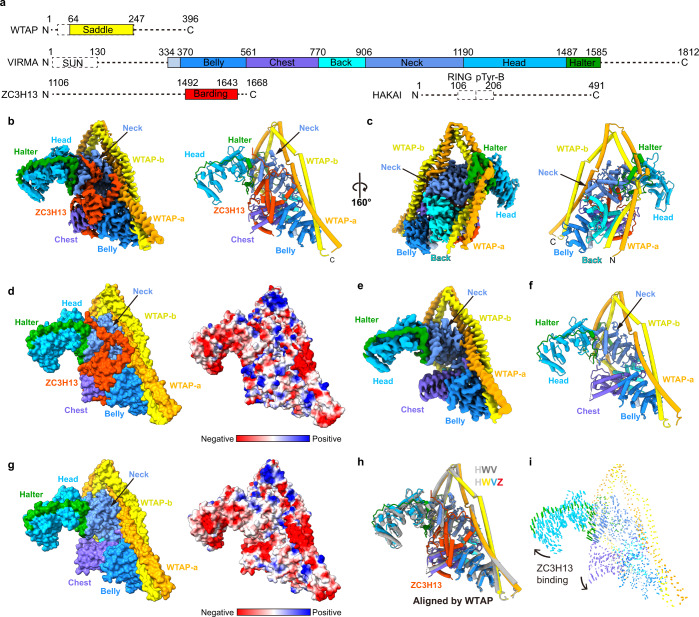
Overview of human MACOM structure. **a** Schematic illustration of the domain organization of proteins in the reconstituted human MACOM complex. The dash lines indicate regions that are invisible in the cryo-EM structure. **b**, **c** The 3.0-Å resolution cryo-EM map (left panel) and cartoon model (right panel) of HWVZ complex in two different views, with WTAP-a in orange, WTAP-b in yellow, ZC3H13 in orange-red, VIRMA Belly domain in dodger-blue, Chest domain in slate-blue, Back domain in cyan, Neck domain in cornflower-blue, Head domain in deep-sky-blue, and Halter domain in green. This color coding applies to the whole article. **d** Surface presentation of the HWVZ complex. The surface in the left panel is colored corresponding to **a**. The surface in the right panel is colored by electrostatic potential. **e** The 3.0-Å resolution cryo-EM map of HWV complex. **f** Overall structure of the HWV complex is shown in the same orientation as the cryo-EM map in **e**. **g** Surface presentation of the HWV complex. The left panel surface is colored corresponding to **a**. The right panel surface is colored by electrostatic potential. **h** Superposition of the HWVZ (colored) and HWV (dark gray) complexes, aligned by WTAP. **i** Conformational change of the HWV complex upon ZC3H13 binding, aligned by WTAP.

To understand the structural basis of the MACOM function, we determined the 3.0-Å resolution structures of the HWVZ and HWV complexes using single-particle cryo-EM analysis (Fig. 2; Supplementary information, Figs. S1, S2). The cryo-EM micrographs and two-dimensional (2D) class averages indicate that the particles adopt different orientations, and 2D class averages show the particles as triangular in shape (Supplementary information, Figs. S1b, S2b). In line with the 2D class averaging analysis, 3D reconstructions display a pyramid-like overall shape, with two V-shaped subunits located at the bottom (Supplementary information, Figs. S1, S2). The quality of the map was sufficient to allow us to build a model from scratch, including well-connected backbones and visible amino acid side chains (Supplementary information, Fig. S3). The model quality was evaluated by three quantitative approaches: MolProbity reflecting model stereochemistry,^25^ per-residue cross-correlation coefficients reflecting model and map agreement,^26^ and Q-scores reflecting structure resolvability^27^ (Supplementary information, Fig. S3 and Table S1).

To our surprise, in the cryo-EM structure of the HWVZ complex, only three components (WTAP, VIRMA, and ZC3H13) can be found, while HAKAI is missing, although it is present in the SDS-PAGE analysis (Fig. 1c), indicating the high flexibility of HAKAI. Human WTAP (residues 64–247), VIRMA (residues 334–1585), and ZC3H13 (residues 1492–1643) form a compact structure shaped like a “warhorse” that represents the core of human MACOM (Fig. 2a–d). Notably, the overall structure of the HWV complex lacking the ZC3H13 component (Fig. 2e–g) is similar to that of the HWVZ complex based on superimposition aligned by WTAP (Fig. 2h), although the horse body is slightly stretched out upon ZC3H13 binding (Fig. 2i; Supplementary information, Video S1). These results suggest that WTAP and VIRMA form the structural core of MACOM with only minor conformational changes upon ZC3H13 binding, and HAKAI is not part of the structural core of MACOM.

### WTAP forms a saddle-shaped homodimer through coiled-coil interactions

**Fig. 3:**
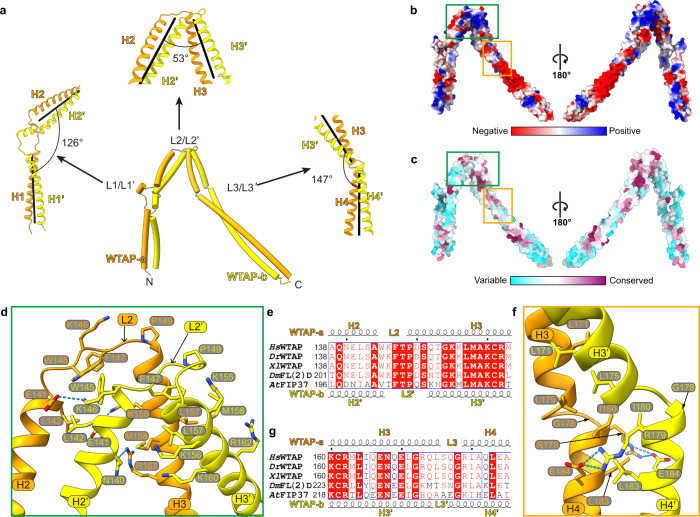
WTAP forms a saddle-shaped homodimer through coiled-coil interaction. **a** Overall structure of WTAP homodimer. The distortion angles of the axes of coiled-coils at L1, L2, and L3 are shown in the left, upper, and right panels, respectively. The axes of coiled-coils are shown as black lines in each panel. **b**, **c** Two different views of the WTAP homodimer surface colored by electrostatic potential (**b**) and sequence conservation (**c**). **d** Intermolecular contacts between WTAP-a and WTAP-b around L2/L2’, corresponding to the green box in **b** and **c**. **e** Sequence alignment of the WTAP homodimer (138–163) containing the L2/L2’ region. *Hs*, *Homo sapiens*; *Dr*, *Danio rerio*; *Xl*, *Xenopus laevis*; *Dm*, *Drosophila melanogaster*; *At*, *Arabidopsis thaliana*. The secondary structure depictions of WTAP-a and WTAP-b are shown in the top and bottom panels. Conserved and similar residues are boxed with red ground and red font, respectively. **f** Intermolecular contacts between WTAP-a and WTAP-b around L3/L3’, corresponding to the orange box in **b** and **c**. **g** Sequence alignment of the WTAP homodimer (160–185) containing the L3/L3’ region, marked as **e**.

Our cryo-EM structure reveals that WTAP forms a homodimer,^22^ with each monomer consisting of four tandem helices (H1–H4/H1’–H4’ with coiled-coil interactions) and three linkers (L1–L3/L1’–L3’) (Fig. 3a). Dimer formation depends on strong monomer–monomer interactions, where the interface area spans ~4620 Å^2^ and contains three salt bridges, eight hydrogen bonds, and extensive non-bonded contacts (Supplementary information, Table S2), as revealed by PDBsum structure bioinformatics analysis.^28^ The dimerization interactions of WTAP are corroborated by XL-MS data (Supplementary information, Fig. S4a, b and Table S3). Interestingly, the two copies of WTAP have a low cross-correlation coefficient (CC = 0.47) between the maps and a low RMSD (15.9 Å) across all 184 pairs of the WTAP dimer, suggesting their conformational heterogeneity. There is a positively charged patch around the H2–L2–H3/H2’–L2’–H3’ turn, which is composed of residues with relatively higher conservation (Fig. 3b–d). A sharp turn was found between H2 and H3/(H2’ and H3’) at a 53° angle (Fig. 3a). Two hydrophobic cores play key roles in the sharp turn formation (Fig. 3d): W145, F147 in L2 and L142 in H2 form a hydrophobic ring around W145 from H2’ coordinating with K155 in H3; the benzene ring of F147 engages in π–π stacking with P149 in L2’ while binding to the hydrophobic patch formed by L157 and M158 in H3’ and L157 in H3. Moreover, several hydrogen bonds in this area also stabilize the sharp turn of H2–L2–H3/H2’–L2’–H3’. These residues are highly conserved in WTAP orthologs (Fig. 3e). The angles between H1/H1’ and H2/H2’ and between H3/H3’ and H4/H4’ are obtuse, being 126° and 147°, respectively, where L1/L1’ between H1/H1’ and H2/H2’ is longer with a short helix that does not form a coiled-coil structure, and L3/L3’ between H3/H3’ and H4/H4’ is very short and contains three residues (Fig. 3a). Although H3/H3’ and H4/H4’ form canonical coiled coils near L3/L3’ (Fig. 3f), residues around L3/L3’ (R173–Q177) are relatively less conserved compared to H3/H3’ and H4/H4’ residues (Fig. 3g). In addition, the two highly conserved glycine residues G172 and G178 reduce the stability of the coiled coil. These sequence patterns provide the basis for L3/L3’ formation. Overall, WTAP forms a saddle-shaped dimer with three unique turns.

### VIRMA adopts a horse-shaped conformation composed of ARM-like (ARML) modules

**Fig. 4:**
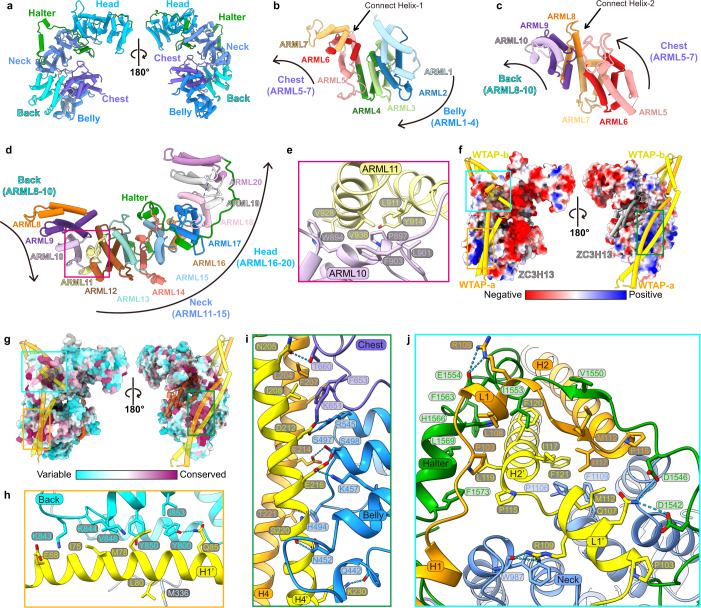
VIRMA adopts a horse-shaped conformation clamped by WTAP homodimer. **a** Structure of VIRMA in two different views. **b**–**d** Structure and packing orientation of different domains in VIRMA. ARML repeats (1–20) are shown in different colors. Curved arrows indicate the inter-repeat packing orientation. Connect helix-1 and -2 separate the Belly, Chest, and Back domains while changing their inter-repeat packing orientation. The noncanonical interaction between ARML10 and 11 separates the Back and Neck domains. Head and Neck domains are divided by Halter domain. **e** Details of the inter-repeat interactions between ARML repeats 10 and 11, corresponding to the magenta box in **d**. **f**, **g** Surface presentation of VIRMA in the HWVZ complex. The surface of VIRMA is colored by electrostatic potential (**f**) and sequence conservation (**g**). ZC3H13 and WTAP are shown in cartoon mode. **h** Intermolecular contacts between WTAP-b (H1’) and Back domain of VIRMA, corresponding to gold box in **f** and **g**. **i** Details of interactions between WTAP H4/H4’ and Belly-Chest domain of VIRMA, corresponding to green box in **f** and **g**. **j** Halter domain tied the WTAP L1/L1’ to the Neck domain of VIRMA. Intermolecular contacts between the WTAP homodimer and VIRMA around L1/L1’, corresponding to the cyan box in **f** and **g**.

VIRMA is the largest component in MACOM and adopts a horse-shaped conformation with 20 ARML modules (Fig. 4a–d). The Belly (ARML1–4) and Chest (ARML5–7) are connected with the same shared helix (Connect Helix-1) (Fig. 4b), and the Chest and Back (ARML8–10) are connected by a similar connection, Connect Helix-2 (Fig. 4c). Inter-repeat packing orientations are changed via Connect Helices (Fig. 4b, c). The Neck (ARML11–15) and Head (ARML16–20) are connected following the canonical stacking architecture of the ARML module (Fig. 4d). Notably, Back and Neck adopt different stacking orientations, mainly due to the hydrophobic interaction between ARML10 and ARML11 (Fig. 4e). Interestingly, a long loop after ARML20 extends along the Head domain and interacts with L1/L1’ of the WTAP homodimer; therefore, it is called the Halter domain (Fig. 4a, d). Although the overall surface charge of VIRMA is mostly negative, several small regions are positively charged (Fig. 4f). These regions are also relatively conserved (Fig. 4g), suggesting that they may be important for interactions with other components of MACOM, especially WTAP.

### WTAP and VIRMA form the structural core of MACOM through extensive interactions

We then analyzed the intermolecular interactions between VIRMA and WTAP dimer. Strong interactions were found: a 2778 Å^2^ interface area with three salt bridges and 15 hydrogen bonds for WTAP-a, as well as a 3303 Å^2^ interface area with three salt bridges and eight hydrogen bonds for WTAP-b (Supplementary information, Table S2). Two positively charged patches in the Back and Belly domains of VIRMA are composed of highly conserved residues and located in the interface with H1/H1’ and H4/H4’ of WTAP, respectively (Fig. 4f, g; Supplementary information, Figs. S5, S6a). Several hydrogen bonds are formed, including Q85 from H1’ of WTAP and Y800 from the Back domain of VIRMA (Fig. 4h), as well as E216 from H4’ of WTAP and S498 from the Belly domain of VIRMA (Fig. 4i). The interactions between the Neck-Halter domain of VIRMA and WTAP are more complicated (Fig. 4j): except hydrophobic interactions of the C-terminal α-helix of the Neck-Halter domain with L1/L1’ and H2/H2’, two hydrogen bonds are formed by conserved residues, including Q107 from L1’ of WTAP and D1546 from the Halter domain of VIRMA, as well as R109 from L1 and E1554 from the Halter domain (Fig. 4j). Moreover, the side chain of R109 from L1’ and the indole ring of W987 from the Neck domain form cation–π interactions in addition to the hydrogen bond between the side chain of R109 and the main chain of W987 (Fig. 4j). These specific interactions allow WTAP and VIRMA to form the MACOM core, and most of the interactions remain in the structure of the HWV complex when ZC3H13 is removed (Fig. 2d, g).

### ZC3H13 stretches the MACOM conformation by interacting with VIRMA

**Fig. 5:**
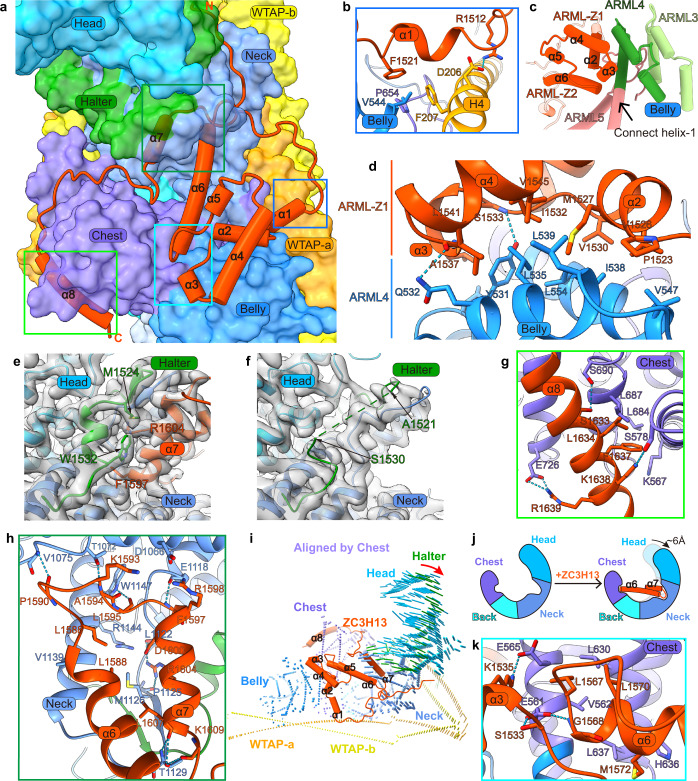
ZC3H13 interacts with VIRMA and induces conformational change of MACOM. **a** Overview of the ZC3H13 Barding domain binding to the HWV complex. ZC3H13 is shown in cartoon mode. The WTAP homodimer and VIRMA are shown in translucent surface. **b** Details of the interface formed by Barding domain-α1, WTAP-a-H4, Chest and Belly domains of VIRMA, corresponding to blue box in **a**. **c** ARML3–4 in Belly domain of VIRMA and ARML-Z1-2 in Barding domain of ZC3H13. ARML repeats of the Belly domain are shown in different colors. ARML repeats Z1 (α2–α4) and Z2 (α5–α6) are colored in orange-red. **d** Inter-repeat interactions of ARML-Z1 in the ZC3H13 Barding domain and ARML-4 in the Belly domain. **e**, **f** Density map of α7 in the Barding domain and Halter domain interface with the corresponding atomic model from the HWVZ and HWV complexes, respectively. **g** Interactions between α8 in the Barding domain and Chest domain of VIRMA, corresponding to the light-green box in **a**. **h** Interactions between α6, α7 of the ZC3H13 Barding domain and Neck domain of VIRMA, corresponding to the forest-green box in **a**. **i** Conformational changes of the HWV complex upon ZC3H13 binding, aligned by the Chest domain of VIRMA. **j** Model of ZC3H13 α6 and α7 binding-induced conformational changes of the HWV complex. **k** Interactions between α3, α6 of the ZC3H13 Barding domain and Chest domain of VIRMA, corresponding to the cyan box in **a**.

Unlike WTAP and VIRMA that form saddle- and horse-like conformations, respectively, the C-terminal fragment of ZC3H13 is only one-tenth of its full length and contains eight α-helices, but it does not exhibit a rigid conformation. Instead, it attaches to the Belly, Chest, and Neck domains of VIRMA and functions like a barding to protect the horse body (Figs. 2a–c, 5a; Supplementary informaiton, Video S1). Therefore, this fragment of ZC3H13 is called the Barding domain. Of particular interest, it is the most conserved fragment in ZC3H13 homologs across species, including flies (Supplementary information, Fig. S6b). The Barding domain of ZC3H13 has a strong interaction with VIRMA, and the interface is ~4435 Å^2^, involving seven salt bridges and 19 hydrogen bonds (Supplementary information, Table S2). In contrast, the interaction between the Barding domain and WTAP is weak, with an interface area of ~256 Å^2^ in which highly conserved residues R1512 and F1521 of the first α-helix of the Barding domain form salt bridges and hydrophobic interactions with D206 and F207 in H4 of WTAP-a, respectively (Fig. 5b). Noteworthily, α2–4 of the Barding domain stack on ARML4 of the Belly domain mainly through hydrophobic interactions in a manner similar to the ARML module (Fig. 5c, d); therefore, α2–4 of ZC3H13 are called ARML-Z1. α5–6 of the Barding domain stack on ARML-Z1 in an ARM-like manner; thus, it is called ARML-Z2 (Fig. 5c). α7 of ZC3H13 interacts with the Neck domain and promotes the formation of a short helix in the Halter domain (Fig. 5e, f). After removing ZC3H13, the cryo-EM density of the short helix of the Halter domain is lost (Fig. 5f). α8, located in the C-terminus of ZC3H13, stacks on the ARML5–7 of the Chest domain mostly through conserved hydrophobic interactions (Fig. 5g). Overall, α1–6 and α8 of ZC3H13 interact with the Belly and Chest domains of VIRMA, while α6 and α7 interact with the Neck domain of VIRMA, thereby pushing the Neck domain away from the Belly and Chest domains (Fig. 5h–k).

### MACOM and MAC assemble into the m^6^A writer complex mainly through WTAP and METTL3 interactions

**Fig. 6:**
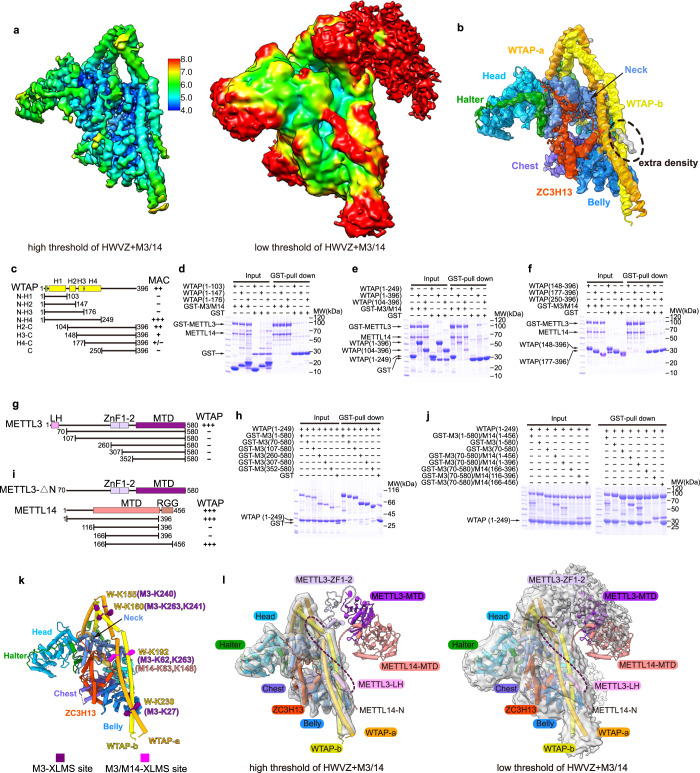
Cryo-EM and crosslinking mass spectrometry analyses of m^6^A writer complex suggest that MACOM interacts with MAC mainly through WTAP and METTL3 interaction. **a** Local resolution map of the HWVZ + M3/14 complex at high and low threshold levels. **b** Structure of the HWVZ complex in the HWVZ + M3/14 cryo-EM map at a high threshold level. The extra density besides the HWVZ complex is marked with a black dotted circle. **c** Schematic view of different WTAP N- and C-terminal truncation constructs. The MAC-binding affinities of these truncations are summarized from **d**–**f** and labeled on the right. **d**–**f** The N- and C-terminal truncation constructs of WTAP were used in GST pull-down assays to assess the interactions with GST-METTL3/METTL14 or mock control GST. **g** Schematic view of different METTL3 N-terminal truncation constructs. The WTAP-binding affinities of these truncation constructs are summarized from **h** and labeled on the right. **h** The N- and C-terminal truncation constructs of GST-METTL3 and mock control GST were used in GST pull-down assays to assess the interactions with WTAP (1–249). **i** Schematic view of different METTL14 N- and C-terminal truncation constructs. The WTAP-binding affinities of MAC formed by these truncation constructs and METTL3 (70–580) are summarized from **j** and labeled on the right. **j** MACs formed by N- and C-terminal truncation constructs of METTL14 and GST-METTL3 (70–580) were used in GST pull-down assays to assess the interactions with WTAP (1–249). It should be noted that there are only two bands in the lane of GST-M3 (70–580)/M14 (166–396) since WTAP (1–249) overlaps with M14 (166–396) on the SDS-PAGE gel. **k** Visible BS3-based MAC-crosslinking residues on WTAP in the structure of the HWVZ complex. METTL3-crosslinking residues are colored in purple. Residues crosslinked with both METTL3 and METTL14 are colored in violet. **l** Model of the m^6^A writer complex in the apo state based on the HWVZ + M3/14 cryo-EM map and biochemistry data. The left panel is the HWVZ + M3/14 cryo-EM map shown in a high threshold level (level = 0.1), and the right panel is the HWVZ + M3/14 cryo-EM map shown in a low threshold level (level = 0.013). Light-coral dash line represents N-terminus of METTL14. Purple and lavender dash lines represent the N-terminus and the linker between Zinc finger and MTA domain of METTL3, respectively.

We assembled the four components of MACOM (HWVZ) with MAC (the METTL3 and METTL14 heterodimer) and obtained a cryo-EM map at 4.4-Å resolution. However, due to its flexibility, the resolution of the MAC region is relatively low (Supplementary information, Fig. S7). At a low threshold (level = 0.013), there are extra densities near L2 and H4 of WTAP in the MACOM–MAC complex compared with the MACOM complex. At a high threshold (level = 0.1), the density near H4 of WTAP becomes clear (Fig. 6a, b; Supplementary information, Fig. S7f), which indicates that H4 of WTAP might play a vital role in MACOM–MAC interactions. Consistently, the GST pull-down results of different WTAP constructs show that H3 and H4 of WTAP are essential for MAC binding (Fig. 6c–f). Furthermore, we analyzed the interactions between MACOM and MAC using XL-MS. Data from bis (sulfosuccinimidyl) suberate (BS3)-based XL-MS indicate that METTL3 might interact with WTAP through its N-terminal LH and Zinc Finger 1–2 (ZF1–2) domains, while METTL14 interacts with all four components mainly through its N-terminal region (Supplementary information, Fig. S4c, d, Table S3 and Video S2). These data are consistent with the results of GST pull-down analysis, demonstrating that the N-terminal LH regions of METTL3 (1–69) and METTL14 (1–115) are essential for their interactions with WTAP (Fig. 6g–j). The XL-MS data provide distance restraints and relative positions of MAC and MACOM. The crosslinked sites of METTL3 on WTAP are located at H3 (K155 and K160) and H4 (K192 and K230), while the crosslinked sites of METTL14 on WTAP are only located at H4 (K192) (Fig. 6k; Supplementary information, Fig. S4c, d and Table S3). Notably, K27 from the N-terminal LH of METTL3 is crosslinked with K230 from H4 of WTAP, suggesting that the extra density near H4 of WTAP may be LH of METTL3 (Fig. 6b, k). The crosslinked sites of METTL3 on VIRMA are located in the Belly domain (K399) and Back domain (K899), while the crosslinked sites of METTL14 on VIRMA are only located in the Back domain (K880, K887, and K899) (Supplementary information, Fig. S4c, d and Table S3). Data from 1-ethyl-3-(3-dimethylaminopropyl) carbodiimide hydrochloride (EDC)-based XL-MS show similar interactions of METTL3 with WTAP and VIRMA, as well as additional interactions between METTL3 and ZC3H13 (Supplementary information, Fig. S4e and Table S3). Taken together, we proposed a functional model of the m^6^A writer complex, including MACOM and MAC subunits, in which METTL3 and METTL14 bind H3 and H4 of WTAP and the Back and Belly domains of VIRMA through the LH domain of METTL3 and the N-terminal fragment of METTL14 (Fig. 6l). In such a way, MACOM can enhance the m^6^A writer activity (Fig. 1g).

### A plausible model of the m^6^A writer complex for RNA substrate binding

**Fig. 7:**
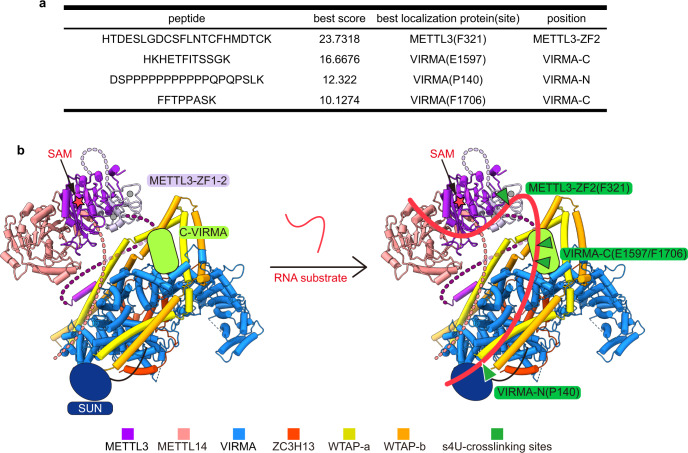
The potential RNA substrate binding model of the m^6^A writer complex. **a** s4U-crosslinking result of MAC/MACOM. **b** Renewed model of m^6^A writer complex in apo state (left panel) and RNA-methylation state (right panel) based on the HWVZ + M3/14 cryo-EM map and biochemistry data. Red star represents SAM. Green triangles in the right panel represent four s4U-crosslinking sites in **a**. Light-coral dash line represents N-terminus of METTL14. Purple and lavender dash lines represent N-terminus and linker between Zinc finger and MTA domain of METTL3, respectively. The models of SUN domain and C-terminus of VIRMA are colored in blue and lime-green, respectively.

To understand the binding mode of RNA substrate by MACOM/MAC complex, the *ACTB-1* RNA oligo is labeled by 4-thiouridine (s4U) and crosslinked with MACOM/MAC complex for mass spectrometry. Four identified s4U-crosslinking sites are located in the ZF2 of METTL3 (F321) and N- and C-terminus of VIRMA (P140, E1597, and F1706) (Fig. 7a; Supplementary information, Fig. S8). The F321 in METTL3 plays a key role in direct interaction with RNA substrate, consistent with previously reported results.^16^ The s4U-crosslinking of VIRMA is further verified by EMSA and methylation assays (Fig. 7a; Supplementary information, Fig. S9a, b). MACOM containing C-terminally truncated VIRMA (1–1586) shows a significant decrease in RNA binding affinity and an almost complete loss of methylation activity (Supplementary information, Fig. S9a, b). Based on the protein–RNA crosslinking data and biochemical results, we propose a possible path of the bound RNA in the m^6^A writer complex (Fig. 7b): MACOM binds the RNA substrate via the N- and C-terminus of VIRMA, enhancing the MAC binding affinity to the RNA substrate (Fig. 1d, h).

## Discussion

### MACOM is a conserved subunit of the m^6^A writer

RNA m^6^A modification is highly conserved in most eukaryotes. METTL3 and METTL14, the two MT-A70 family proteins in the catalytic core of the m^6^A writer, are also highly conserved from yeasts to humans. In addition to the catalytic core components of MAC, more components of MACOM have been identified in higher animals and plants, suggesting that more regulation is required for m^6^A modification in complicated biological systems.^29^ Although the components of MACOM are not widely present, WTAP co-evolves with the METTL3 and METTL14 complex in most eukaryotic species.^29^ VIRMA, ZC3H13, and HAKAI co-exist with WTAP in metazoans.^18^ Interestingly, only VIRMA and HAKAI homologs are co-purified with WTAP in multicellular plants, while ZC3H13 has not been identified in plants by biochemical purification^21^ or protein sequence BLAST (data not shown). Our structural study shows that ZC3H13 uses its C-terminal Barding domain to interact with VIRMA and induce the conformational change of MACOM (Fig. 5). It is possible that another protein different from ZC3H13 plays a similar role in multicellular plants.

### MACOM is required for efficient m^6^A modification

Recent study reported that WTAP homologue Fl(2)d is present as homodimer through the region (125–536).^22^ The WTAP dimer region (65–245) in our structure is corresponding to the Fl(2)d region (127–308) (Supplementary information, Fig. S6a), which is fitted well with previous biochemical results. Based on our structural study, WTAP and VIRMA constitute the MACOM core and form a warhorse-like shape, and ZC3H13 binds to VIRMA through its C-terminal Barding domain. The catalytic core component of MAC, METTL3, interacts with MACOM mainly through WTAP, as demonstrated by the XL-MS results showing that more than half of the crosslinking sites of METTL3 are related to WTAP. In vitro RNA substrate binding and m^6^A methyltransferase activity assays demonstrate that MACOM (HWVZ and WVZ) can directly bind to RNA substrates and significantly increase the m^6^A writer activity (Fig. 1). Specifically, removing ZC3H13 dramatically reduces substrate binding and methyltransferase activity (Fig. 1d, g), suggesting that ZC3H13 is a key component of MACOM for substrate binding and m^6^A writer activity. Furthermore, based on the protein–RNA crosslinking data and biochemical results, we propose a plausible model of the m^6^A writer complex for RNA substrate binding in which MACOM and MAC bind different regions of the RNA substrate (Fig. 7). Previous study demonstrated that VIRMA in MACOM mediates preferential m^6^A mRNA methylation in 3′ UTR and near stop codon through association with polyadenylation cleavage factors CPSF5 and CPSF6 in an RNA-dependent manner.^10^ In addition, RBM15, another key component of MACOM, was shown to play an important role in binding the specific site of RNA substrate and recruit m^6^A writer complex.^7,9,30^ Further studies of MACOM–MAC complex containing RBM15 would help to gain further insights into the mechanism of substrate selection of m^6^A writer complex.

Although HAKAI is absent in our cryo-EM structures, HAKAI is co-purified with the other three components in gel filtration (Fig. 1b, c) and contacts them using its pTyr-B domain based on the BS3-XL-MS data (Supplementary information, Fig. S4b, d and Table S3). Notably, the structure of HWVZ complex is fitted well in negative staining EM map of WVZ complex (Supplementary information, Fig. S10). These results indicate that HAKAI is not missing but flexible in the HWVZ complex, which is consistent with the observation that the N-terminal SUN domain of VIRMA, a recently reported HAKAI-interacting region, is also absent in our cryo-EM structures.^22^ Our RNA binding and enzymatic activity assays indicate that HAKAI is not crucial for the substrate binding and m^6^A modification in vitro (Fig. 1d, g), which might be caused by the lack of potential phosphorylation and ubiquitylation that may be important for HAKAI function. According to recent studies, HAKAI is essential for MACOM stability and maintenance of m^6^A levels in vivo.^22,23^ Therefore, further studies are required to assess the role and molecular mechanism of HAKAI in MACOM.

### Structures of the MACOM core provide a basis for the regulation of m^6^A modification in human diseases

It has been proven that m^6^A modification is involved in various biological processes, and deregulation of m^6^A modification leads to a variety of human diseases. The most conserved core component of MACOM, WTAP, was discovered due to its relationship with Wilms’ tumor 1 (WT1) and was named as Wilms’ tumor 1-associated protein.^31^ WTAP has recently been shown to be upregulated in many tumors, such as acute myeloid leukemia.^32,33^ Our cryo-EM structures, especially the high-resolution structure of the MACOM core, provide a basis for the development of inhibitors or drugs targeting m^6^A modification to achieve potential therapeutic efficacy for human diseases such as cancers.^24^

## Materials and methods

### Cell lines and culture

*Escherichia coli* (*E. coli*) BL21 (DE3) (Novagen) and DH10Bac (Thermo Fisher Scientific) were grown in LB. Two kinds of insect cell lines, *Spodoptera frugiperda* (Sf9, Expression systems) cells and *Trichoplusia ni* (High Five, Expression systems) cells, were used for virus preparation and recombinant protein expression, respectively. Sf9 and High Five cells were grown at 27 °C and 130 rpm in SIM SF expression medium and SIM HF expression medium (Sino Biological), respectively.

### Protein construction, expression, and purification

The cDNAs of full-length METTL3, METTL14, HAKAI, and partial ZC3H13 (residues 1106–1668) were subcloned into modified or unmodified pFastBac-HTb vectors, resulting in the expression of N-GST-TEV-METTL3, N-6× His-TEV-METTL14, untagged HAKAI, and N-Twin-Strep-tag-ZC3H13 (1106–1668). The cDNAs of full-length WTAP and VIRMA were subcloned into the pFastBac-Dual vector, resulting in co-expression of N-6× His-WTAP and N-6× His-VIRMA. MAC (METTL3/METTL14) or four components of the MACOM complex, HAKAI/WTAP/VIRMA/ZC3H13 (HWVZ), were co-expressed in High Five insect cells, respectively. About 60 h after P2 virus infection at 27 °C, cells were harvested by centrifugation at 4000 rpm for 10 min and cell pellets were collected for protein purification.

Cells were resuspended in Buffer A (150 mM KCl, 20 mM Tris-HCl, pH 8.0, 10% glycerol, 25 mM imidazole) with 0.5 mM PMSF and protease inhibitors, lysed by adding 0.5% Triton X-100 and shaken gently for 20 min at 4 °C. After centrifugation at 18,000 rpm for 60 min, the supernatant was collected for further purification. The METTL3/METTL14 complex was purified by tandem affinity chromatography of Ni-NTA and GST. After removal of His- and GST-tags by TEV protease digestion at 4 °C overnight, the protein solution was further purified by a Heparin HP column. The concentrated solution was loaded onto a Superdex 200 Increase 10/300 GL column (GE Healthcare cytiva) equilibrated in SEC buffer (100 mM KCl, 20 mM HEPES, pH 7.5, 10% glycerol, 1 mM DTT). Fractions of the METTL3/METTL14 complex were collected and concentrated to 20 mg/mL. The purification procedures of METTL3 and METTL14 truncations were the same as those of the METTL3/METTL14 complex. The HWVZ complex was purified by tandem affinity chromatography of Ni-NTA and STREP beads. The concentrated solution was loaded onto a Superose 6 Increase 10/300 GL column equilibrated in SEC buffer. Fractions corresponding to the HWVZ complex were collected and concentrated to ~10 mg/mL. The purification procedures of HAKAI/WTAP/VIRMA (HWV), WTAP/VIRMA/ZC3H13 (WVZ), and WTAP/VIRMA (WV) complexes were the same as those of the HWVZ complex.

The cDNAs of truncated WTAP constructs were cloned into pET28a (with a 6× His-SUMO tag) for protein expression in *E. coli* BL21 (DE3). When *E. coli* were cultured at 37 °C to an OD600 of 0.6, isopropyl β-D-1-thiogalactopyranoside (IPTG) was added to a final concentration of 0.2 mM. Then *E. coli* were grown for ~16 h at 18 °C. The cells were collected by centrifugation, resuspended in buffer containing 20 mM Tris-HCl, pH 8.0, 100 mM KCl, 25 mM imidazole, and lysed with high-pressure homogenization. The truncated WTAP constructs were first purified by Ni-NTA affinity chromatography. Then protease ULP1 was added to remove the 6× His-SUMO-tag, and dialysis was applied to remove imidazole. Then the sample was subjected to the second Ni-NTA chromatography, and the flow-through was collected. The concentrated solution was loaded onto a Superdex 75 HiLoad 16/60 column (GE Healthcare) equilibrated in SEC buffer. Fractions corresponding to truncated WTAP constructs were collected and concentrated to ~20 mg/mL.

### Sample preparation for cryo-EM

To prepare HWVZ or HWV complex samples for cryo-EM study, the HWVZ, HWV or HWVZ + M3/M14 complex was crosslinked by 0.5 mM bis(sulfosuccinimidyl)suberate (BS3, Thermo Fisher) for 2 h at 25 °C and quenched with 20 mM glycine. The complex was further treated by density gradient centrifugation. The gradient was generated from a light solution containing 10% (v/v) glycerol, 20 mM HEPES, pH 7.5, 100 mM KCl, 1 mM DTT, and a heavy solution containing 30% (v/v) glycerol, 20 mM HEPES, pH 7.5, 100 mM KCl, 1 mM DTT. Centrifugation was performed at 38,000 rpm in a Beckman SW41Ti swinging bucket rotor for 16 h at 4 °C. The peak fractions were collected and further dialyzed with buffer containing 100 mM KCl, 20 mM HEPES, pH 7.5, and 1 mM DTT at 4 °C overnight. The crosslinked HWVZ or HWV complex was concentrated to 1.0 mg/mL, and then applied to cryo-EM grids.

### Preparation of RNA substrate

The RNA substrate *ACTB-1* from the stop codon region of human *ACTB* mRNA containing two m^6^A sites and one RBM15 binding site^9^ (5′- GGCCCCUCCAUCGUCCACCGCAAAUGCUUCUAGGCGGACUAUGACUUAGUUGCGUUACACCCUUUCUUGACAAAACCUAACUUGCACAGAAAACA-3′) was obtained by in vitro transcription using T7 RNA polymerase. A linearized recombinant pUC19 plasmid containing the target sequence with 5′-hammerhead ribozyme sequence and free 3′-end digested by *Eco*RI was used as the DNA template for in vitro transcription of *ACTB-1* RNA substrate. In vitro transcription was performed at 37 °C for 8 h in the reaction buffer containing 100 mM HEPES, pH 7.9, 10 mM MgCl_2_, 10 mM DTT, 6 mM NTP each, 2 mM Spermidine, 200 μg/mL linearized plasmid, and 100 μg/mL T7 RNA polymerase. The transcripts were purified by 8% denaturing urea PAGE, eluted from gel slices and precipitated with isopropanol. After centrifugation, the RNA precipitant was collected, washed twice with 70% ethanol and air-dried, and the RNA was dissolved in ultrapure water. FAM-labeled *ACTB-1* RNA was produced by Silencer^®^ siRNA Labeling kit-FAMTM according to the product instructions.

### EMSA

An aliquot of 5 nM FAM-labeled *ACTB-1* RNA was mixed with increasing concentrations of protein from 0.25 to 256 times of RNA concentration with a total of 11 gradients in 10 μL buffer containing 20 mM HEPES, pH 7.5, 100 mM KCl, 10% glycerol, 1 mM DTT and incubated at 25 °C for 20 min. Electrophoresis was performed with 8% native PAGE gels in running buffer containing 1× Tris-borate-EDTA (TBE) buffer at 4 °C for 2.5 h. Gels were scanned using a Typhoon FLA-9000 (GE Healthcare). As the complexes of RNA and MACOM cannot run into gels and were held as black deposits in the gel-loading wells due to their biochemical properties, we quantified the RNA-binding ratios by measuring the reduction of free RNA. Free RNA was quantified using ImageJ. Binding curves were fit individually using GraphPad Prism 9.0 software fitting with “One site–Specific binding with Hill slope” (GraphPad Software). Curves were normalized as the percentage of bound oligonucleotides and reported as the means ± SD of the interpolated *K*_d_ from three independent experiments.

### BS3/EDC-mediated crosslinking mass spectrometry

The purified complexes were incubated with 0.5 mM BS3 (Thermo Fisher Scientific, 21580) in reaction buffer containing 50 mM HEPES, pH 7.5, 100 mM KCl, and 5% glycerol at 25 °C for 2 h or 5 mM EDC (Thermo Fisher Scientific, PG82073) in reaction buffer containing 50 mM HEPES, pH 7.2, 100 mM KCl, and 5% glycerol at 25 °C for 3 h. Crosslinked complexes were further purified to remove oligomers and glycerol by size exclusion chromatography. The proteins (10 μg) were precipitated and digested for 16 h at 37 °C by trypsin at an enzyme-to-substrate ratio of 1:50 (w/w). The trypsin-digested peptides were desalted and loaded on an in-house packed capillary reverse-phase C18 column (40-cm length, 100-µM ID × 360-µM OD, 1.9-µM particle size, 120-Å pore diameter) connected to an Easy LC 1200 system. The samples were analyzed with a 120-min HPLC gradient from 6% to 35% of buffer B (buffer A: 0.1% formic acid in water; buffer B: 0.1% formic acid in 80% acetonitrile) at 300 nL/min. The eluted peptides were ionized and directly introduced into a Q-Exactive mass spectrometer using a nano-spray source. Survey full-scan MS spectra (300–1800 m/z) were acquired in the Orbitrap analyzer with resolution *r* = 70,000 at 400 m/z. Crosslinked peptides were identified and evaluated using pLink2 software.^34^

The acquired MS/MS data were analyzed against a homemade database (including all the target proteins downloaded from UniProt) using pLink2. Cysteine alkylation by iodoacetamide was specified as a fixed modification with a mass shift of 57.02146 and methionine oxidation as a variable modification. Precursor mass tolerance and fragment mass tolerance were 20 p.p.m. BS3 (crosslinking sites K and protein N-terminus, xlink mass shift 138.0680796, mono-link mass shift 156.0786442) was defined as a crosslinker. Peptide length was set between 6 amino acids and 60 amino acids. Maximum missed cleavage sites were three. False discovery rate was set as 5%. We screened crosslinking sites that had at least 3 spectra with *E*-values ≤ 0.01 for structural analysis.

### GST pull-down assay

GST pull-down assay was performed to detect protein–protein interactions using GST-tagged or untagged proteins purified from bacterial or insect cells. First, GST-tagged protein and untagged protein were mixed and incubated on ice for 30 min. Then the protein mixture was incubated with 15 μL GST beads in a total volume of 500 μL in binding buffer (100 mM KCl, 20 mM HEPES, pH 7.5, 10% glycerol, 1 mM DTT) at 4 °C for 2 h with gentle rotation. After centrifugation at 300× *g* for 3 min, the supernatant was removed, and the beads were washed three times using wash buffer (100 mM KCl, 20 mM HEPES, pH 7.5, 10% glycerol, 1 mM DTT, 0.1% NP-40). Another three washes were performed using binding buffer, followed by SDS-PAGE analysis.

### In vitro m^6^A methyltransferase activity assay

In vitro methyltransferase activity assay was performed as previously published^12^ with several modifications. Briefly, the 50 μL reaction mixture contained 1 μM RNA substrate, 10 nM MAC, 15 nM MACOM, 30 μM d3-SAM, 100 mM KCl, 50 μM ZnCl_2_, 0.01% Triton X-100, 20 mM Tris, pH 7.5, 20 μg/μL BSA, 5 mM DTT, and 0.2 U/μL RRI (Recombinant RNase Inhibitor, Takara). The reaction was carried out by incubating at 37 °C for 1 h and quenched by inactivating the enzyme at 95 °C for 5 min. RNA was digested at 42 °C for 2 h with 20 mM CH_3_COONH_4_ and 1 U nuclease P1 (Wako). Afterwards, 1 μL BAP (Bacterial Alkaline Phosphatase, TOYOBO) and 6.4 μL 10× BAP Buffer were added, and the reaction was incubated at 37 °C for 2 h. The sample was diluted to 120 µL and then filtered through a 0.22-μm filter (Millipore). A 5-μL aliquot of the sample was injected into the LC/MS/MS system. The nucleosides were separated by reverse phase ultra-performance liquid chromatography on a C18 column with online MS detection using an Agilent 6460 QQQ triple-quadrupole LC mass spectrometer in positive electrospray ionization mode. The nucleosides were quantified by using nucleoside-to-base ion mass transitions of 282 to 150 (d3-m^6^A) and 268 to 136 (A). Quantification was performed in comparison with the standard curve obtained from pure nucleotide standards running on the same batch of samples. The ratios of d3-m^6^A to A and d3-m^6^A to probe were calculated on the basis of the calibrated concentrations.

### UV crosslinking mass spectrometry of s4U-ACTB-1 and m^6^A writer complex

The purified m^6^A writer complex was incubated with s4U-ACTB-1 for 30 min on ice to form the stable complex. Then the sample was UV-crosslinked on ice with 3 J/cm^2^ at 365 nm wavelength with a UVP-crosslinker (analytik jena). Subsequently, the sample was treated with RNase I/RNase A/RNase T1 mixture at 1 U/μL concentration for 1 h at 37 °C. Proteins were precipitated with acetone. The protein pellet was dried by using a SpeedVac for 1−2 min. The pellet was subsequently dissolved in 8 M urea, 100 mM Tris-HCl, pH 8.5. TCEP (final concentration is 5 mM) (Thermo Scientific) and Iodoacetamide (final concentration is 10 mM) (Sigma) for reduction and alkylation were added to the solution and incubated at room temperature for 30 min, respectively. The protein mixture was diluted four times and digested overnight with trypsin at 1:50 (w/w) (Promega). The digested peptide solutions were desalted using a MonoSpinTM C18 column (GL Science, Tokyo, Japan) and dried with a SpeedVac. The peptide mixture was analyzed by a homemade 30 cm-long pulled-tip analytical column (75 μm ID packed with ReproSil-Pur C18-AQ 1.9 μm resin, Dr. Maisch GmbH, Germany), the column was then placed in-line with an Easy-nLC 1200 nano HPLC (Thermo Fisher Scientific) for mass spectrometry analysis. The analytical column temperature was set at 55 °C during the experiments. The mobile phase and elution gradient used for peptide separation were as follows: 0.1% formic acid in water as buffer A and 0.1% formic acid in 80% acetonitrile as buffer B, 0–1 min, 5%–8% B; 1–114 min, 8%–35% B; 114–115 min, 35%–50% B; 115–116 min, 50%–100% B; 116–120 min, 100% B. The flow rate was set as 300 nL/min.

Data-dependent tandem mass spectrometry (MS/MS) analysis was performed with an Orbitrap Eclipse mass spectrometer (Thermo Fisher Scientific). Peptides eluted from the LC column were directly electrosprayed into the mass spectrometer with the application of a distal 2-kV spray voltage. The cycle time was set to 3 s. Full scan resolution was set to 120,000 and MS/MS scan resolution was set to 30,000 with isolation window of 1.6 m/z. The dynamic exclusion settings used were as follows: charge exclusion, 1, and > 7; exclude isotopes, on; and exclusion duration, 30 s. MS scan functions and LC solvent gradients were controlled by the Xcalibur data system (Thermo Fisher Scientific).

The acquired MS/MS data were analyzed by RNPxl^35^ using 10 ppm for MS1. The s4U-crosslinking peptides (mass adduct is same as U-H_2_O). Raw data were searched against a FASTA database consisting of the protein sequences of the proteins in the complexes using RNPxl node combined in PD software with 0.01 FDR. The result is filtered by score (> 5) and the MS2 spectrum. MS2 spectra are annotated in TOPPView.^36^

### Cryo-EM data acquisition

The samples of HWVZ, HWV, and HWVZ + M3/14 complexes were diluted at a final concentration of ~1 mg/mL. Three microliters of the samples were applied onto glow-discharged 200-mesh R2/1 Quantifoil copper grids. The grids were blotted for 4 s and rapidly cryocooled in liquid ethane using a Vitrobot Mark IV (Thermo Fisher Scientific) at 4 °C and 100% humidity. The samples were imaged in a Titan Krios cryo-electron microscope (Thermo Fisher Scientific) operated at 300 kV with a GIF energy filter (Gatan) at a magnification of 105,000× (corresponding to a calibrated sampling of 0.82 Å per pixel) for three samples. Micrographs were recorded by EPU software (Thermo Fisher Scientific) with a Gatan K3 Summit direct electron detector, where each image was composed of 30 individual frames with an exposure time of 2.5 s and an exposure rate of 22.2 electrons/s/Å^2^. A total of 12,745 movie stacks for HWVZ, 7642 movie stacks for HWV, and 14,168 movie stacks for HWVZ + M3/14 were collected.

### Single-particle image processing and 3D reconstruction

All micrographs were first imported into Relion^37^ for image processing. The motion-correction was performed using MotionCor2^38^ and the contrast transfer function (CTF) was determined using CTFFIND4.^39^ Then the micrographs with “rlnCtfMaxResolution < 5” were selected using the “subset selection” option in Relion. All particles were autopicked using the NeuralNet option in EMAN2.^40^ Then, particle coordinates were imported to Relion, where the poor 2D class averages were removed by several rounds of 2D classification. The initial maps were built and classified using the ab initio 3D reconstruction option in cryoSPARC^41^ without any symmetry applied. The 3D homogeneous refinements, local and global CTF refinements, and non-uniform refinements were performed using the selected 395,916 particles for the HWVZ complex, 282,821 particles for the HWV complex, and 199,741 particles for the HWVZ + M3/14 complex, resulting in ~3.0-Å, ~3.0-Å, and ~4.4-Å resolution maps, respectively. The resolution for the final maps was estimated with the 0.143 criterion of the Fourier shell correlation curve. Resolution map was calculated in cryoSPARC using the “Local Resolution Estimation” option. The figures were prepared using UCSF Chimera^42^ or UCSF ChimeraX^43^ (see more information in Supplementary information, Figs. S1–S3, S7 and Table S1).

### Model building

Model building was first performed based on the 3.0-Å resolution cryo-EM map of the HWVZ complex. As none of these molecules has structural information, de novo model building was conducted. Phenix.map_to_model^9^ was first used to generate the initial model. Coot^44^ was then used to confirm the amino acid sequence registration of the initial model and assign amino acids to the cryo-EM density regions that were not resolved by phenix.map_to_model. Notably, the assignment of amino acid sequence by Coot was based on bulky residues (Trp, Lys, Arg, Phe, and Tyr). An atomic model composed of WTAP (residues 64–247), VIRMA (residues 334–1585), and ZC3H13 (residues 1492–1643) was obtained. The resulting model was refined using phenix.real_space_refine.^45^

To build the atomic model for the HWV complex, the final model of the HWVZ complex without the ZC3H13 subunit was fitted into the 3.0-Å resolution cryo-EM map of the HWV complex, followed by the optimization using phenix.real_space_refine and Coot. The model quality was evaluated by MolProbity^25^ and Q-scores.^27^ Statistics of the map reconstruction and model optimization are summarized in Supplementary information, Table S1. PDBsum structure bioinformatics^28^ was used to identify the key residues involved in interactions between subunits in our structures. All figures were made using UCSF Chimera or UCSF Chimera X.

### Quantification and statistical analysis

For the quantification of the binding affinities of the HWVZ, WVZ, HWV, and WV complexes with FAM-labeled *ACTB-1* RNA (Fig. 1d), measurements were carried out using ImageJ. GraphPad Prism was used to perform the statistical analysis of measuring results. Each data point represents the average of three independent experiments. Error bars represent SD. For the quantification of *ACTB-1* RNA *N*^6^-adenosine methylation activity (Fig. 1g), GraphPad Prism was used to perform the statistical analysis of measuring results. Each data point represents the average of two independent experiments. Error bars represent SD.

### Negative staining analysis

The purified crosslinked WVZ complex was applied to Superose 6 Increase 10/300 GL column (GE Healthcare) equilibrated with the gel filtration buffer. The complex was diluted to 35 ng/μL with SEC buffer. Five microliters of sample were applied to the glow-discharged 200 mesh carbon-coated copper grids. The samples were stained using 0.75% uranyl formate and air-dried. Data were collected on a Talos L120C transmission electron microscope equipped with a 4K × 4K CETA CCD camera (Thermo Fisher Scientific). Images were recorded at a nominal magnification of 92,000×, corresponding to a pixel size of 1.55 Å. CTF parameters of each micrograph were determined using CTFFIND4.^39^ Particles were picked and subjected to two rounds of 2D classification, followed by de novo 3D model generation, 3D classification, and auto-refinement in Relion.^37^

## Supplementary information

Supplementary information, Figure S1

Supplementary information, Figure S2

Supplementary information, Figure S3

Supplementary information, Figure S4

Supplementary information, Figure S5

Supplementary information, Figure S6

Supplementary information, Figure S7

Supplementary information, Figure S8

Supplementary information, Figure S9

Supplementary information, Figure S10

Supplementary information, Table S1

Supplementary information, Table S2

Supplementary information, Table S3

Supplementary information, Video S1

Supplementary information, Video S2

Supplementary information, Video legend

## Data Availability

Cryo-EM maps of the HWVZ, HWV, and HWVZ + M3/14 complexes in this study have been deposited in the wwPDB OneDep System under EMD accession codes EMD-31946, EMD-31947, and EMD-34169, respectively. The associated atomic models of HWVZ and HWV complexes are under PDB codes 7VF2 and 7VF5, respectively. The crosslinking mass spectrometry data have been deposited in ProteomeXchange with the primary accession code IPX0003503000.
