# Supplementary material for: Cryo-EM structures of human m6A writer complexes
Source: Cell Res. 2022 Sep 27;32(11):982–94. doi: 10.1038/s41422-022-00725-8 (PMC9652331; doi:10.1038/s41422-022-00725-8)
Supplement: Supplementary file 2 — Supplementary information, Figure S2 [file 41422_2022_725_MOESM2_ESM.pdf]

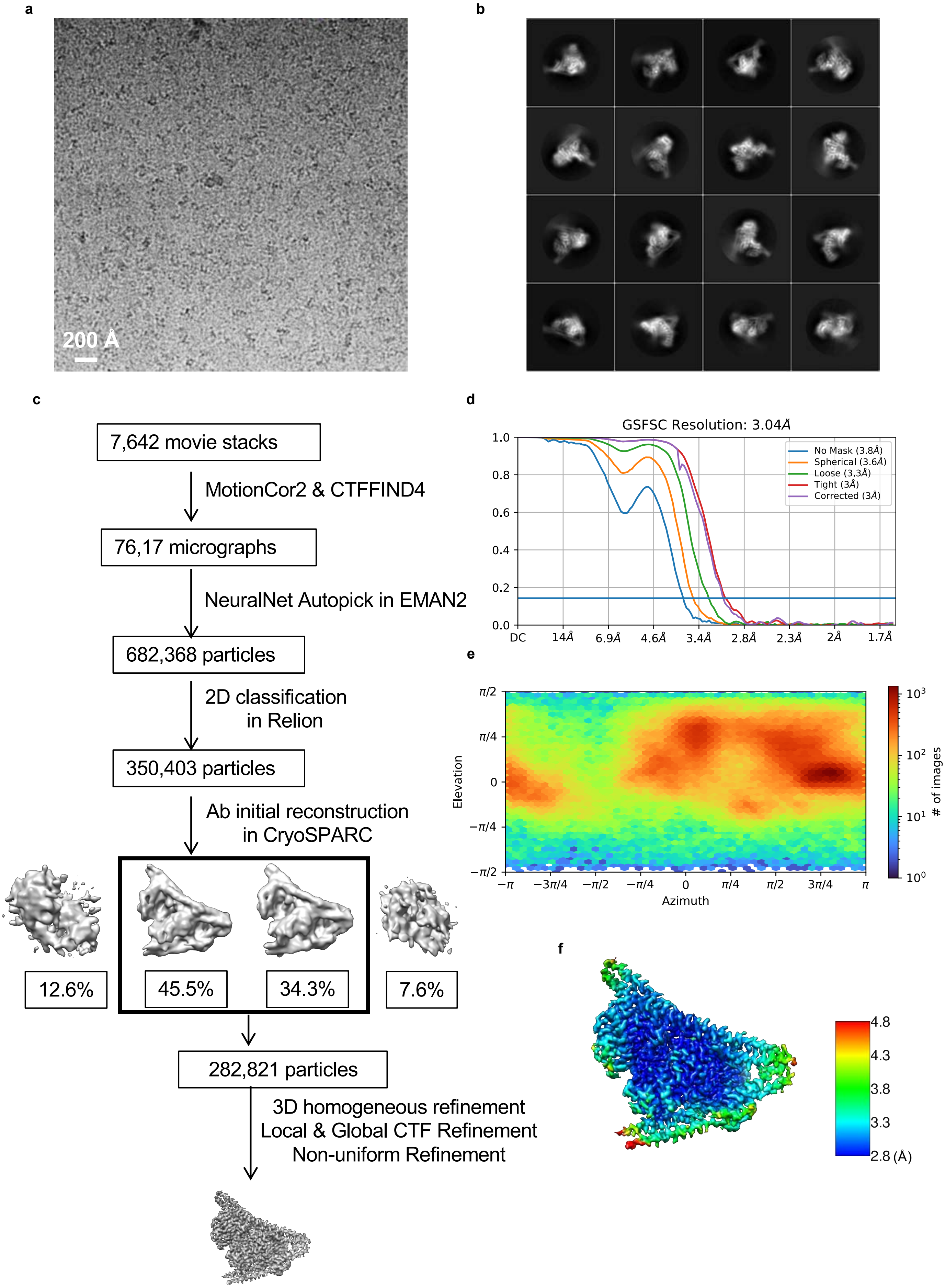

Supplementary information, Fig. S2. Single-particle cryo-EM analysis of the HWV complex. a Representative motion-corrected cryo-EM micrograph. **b** Reference-free 2D class averages. **c** Workflow of the data processing. **d** Gold standard FSC plots calculated in cryoSPARC. **e** Euler angle distribution of theparticle images. **f** Resolution map for the final 3D reconstruction.
