# Supplementary material for: Cryo-EM structures of human m6A writer complexes
Source: Cell Res. 2022 Sep 27;32(11):982–94. doi: 10.1038/s41422-022-00725-8 (PMC9652331; doi:10.1038/s41422-022-00725-8)
Supplement: Supplementary file 3 — Supplementary information, Figure S3 [file 41422_2022_725_MOESM3_ESM.pdf]

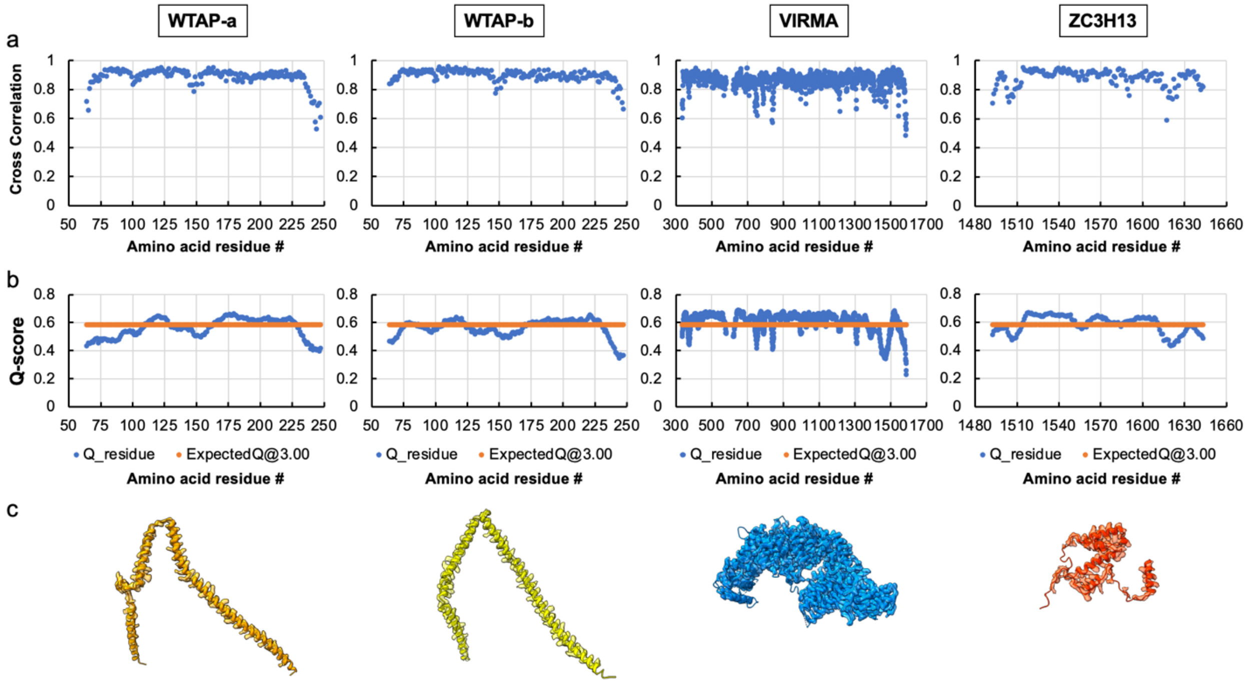

Supplementary information, Fig. S3. Model validation of VIRMA, WTAP-a, WTAP-b, and ZC3H13 subunits. a Per-residue cross-correlation coefficient between the model and 3-Å map. **b** Q-score for each amino acid residue in model and 3-Å map; the orange line represents the expected Q-score at 3-Å resolution (0.5862) based on the correlation between Q-scores and map resolution. **c** Individual cryo-EM densities of two monomers of WTAP, VIRMA, and ZC3H13 from the 3-Å map.
