# Supplementary material for: Cryo-EM structures of human m6A writer complexes
Source: Cell Res. 2022 Sep 27;32(11):982–94. doi: 10.1038/s41422-022-00725-8 (PMC9652331; doi:10.1038/s41422-022-00725-8)
Supplement: Supplementary file 4 — Supplementary information, Figure S4 [file 41422_2022_725_MOESM4_ESM.pdf]

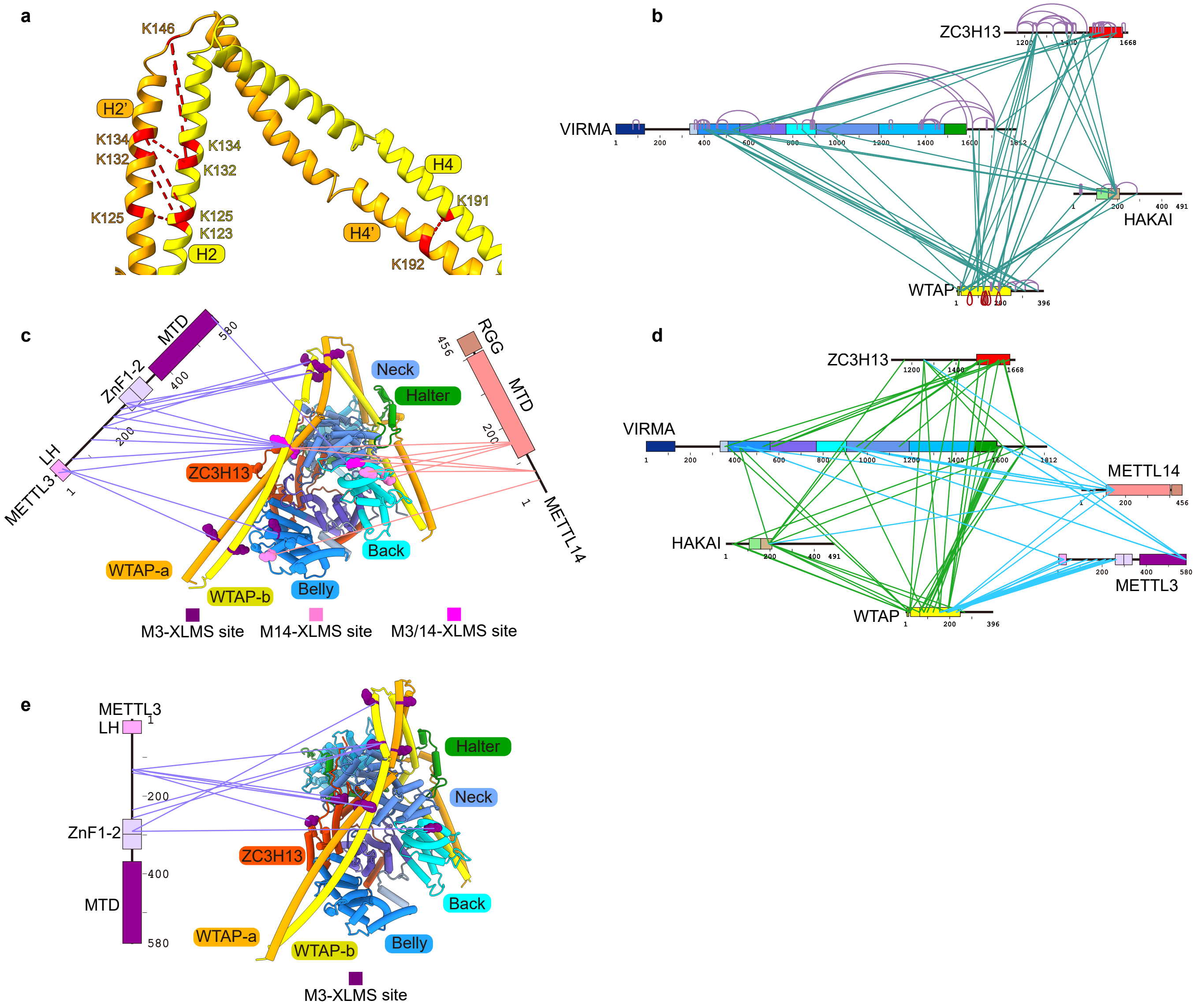

Supplementary information, Fig. S4. XL-MS analysis of MACOM and m^{6}A writer complex. a Intermolecular cross-linking sites of the WTAP homodimer. The paired cross-linking residues are colored in red and linked by a red dash line. **b** Schematic summary of BS3-cross-linking residues identified inter- and intra-molecular contacts in HWVZ complex. The intra- and inter- molecular cross-linking residues are shown as solid line colored in medium-purple and sea-green, respectively. **c** The MAC-cross-linking residues with BS3 in the cryo-EM structure of the HWVZ complex. METTL3-cross-linking residues are colored in purple. METTL14-cross-linking residues are colored in pink. Residues cross-linked with both METTL3 and METTL14 are colored in violet. Schematics of METTL3 and METTL14 are shown on the left and right, respectively. Residues cross-linked with METTL3 and METTL14 are linked to the corresponding positions of METTL3 and METTL14. **d** Schematic summary of BS3-cross-linking residues that identified inter-molecular contacts in the m^{6}A writer complex. The intermolecular cross-linking residues inside HWVZ complex are shown as solid line and colored in green, while those between METTL3/METTL14 and the HWVZ complex are colored in deep-sky-blue. **e** The MAC-cross-linking residues with EDC in the cryo-EM structure of the HWVZ complex. METTL3-cross-linking residues are colored in purple. The schematic of METTL3 is shown at the left.
