# Supplementary material for: Cryo-EM structures of human m6A writer complexes
Source: Cell Res. 2022 Sep 27;32(11):982–94. doi: 10.1038/s41422-022-00725-8 (PMC9652331; doi:10.1038/s41422-022-00725-8)
Supplement: Supplementary file 5 — Supplementary information, Figure S5 [file 41422_2022_725_MOESM5_ESM.pdf]

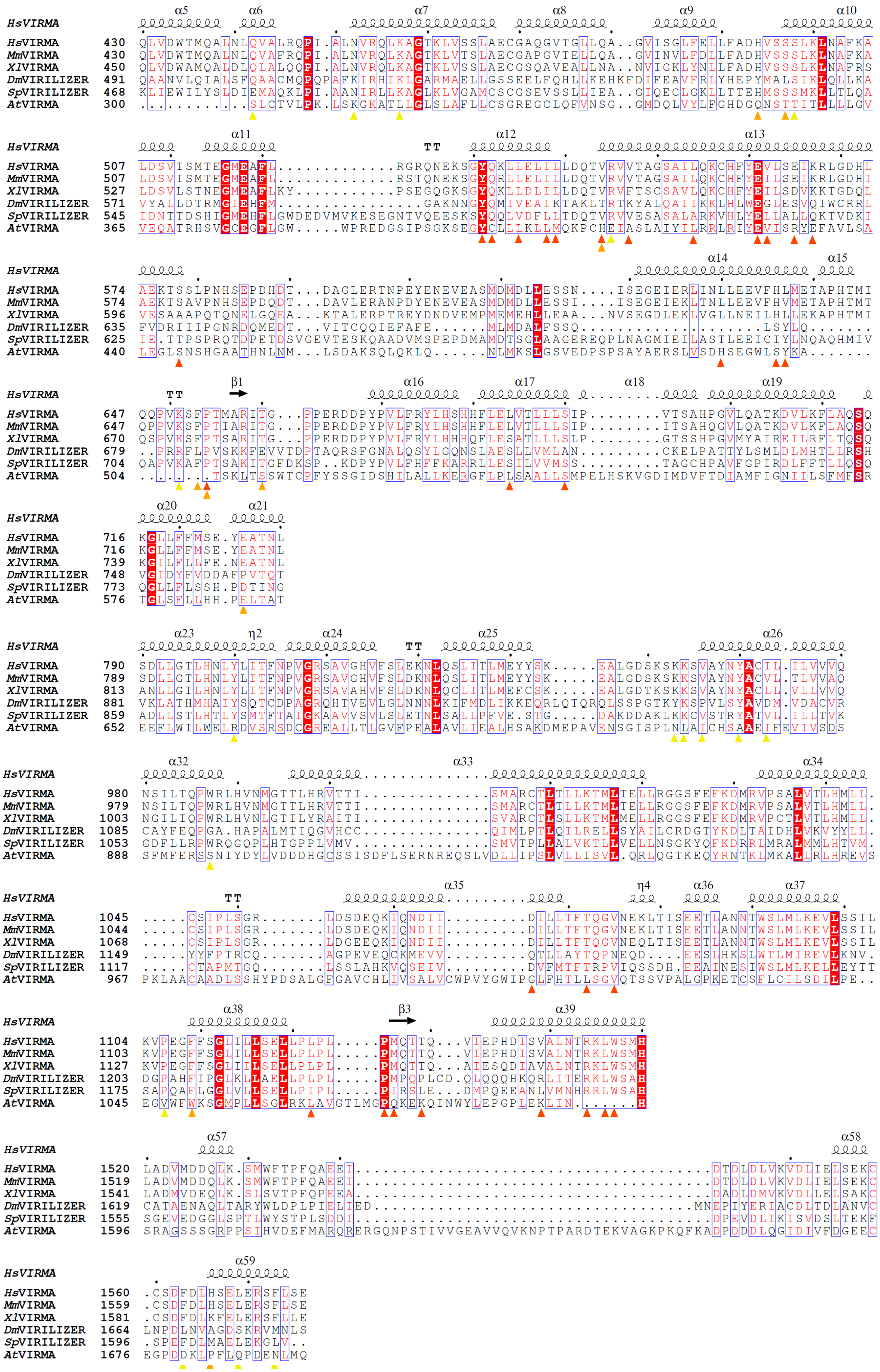

Supplementary information, Fig. S5. Comparison of sequences (*Hs, Homo sapiens; Dr, Danio rerio; Xl, Xenopus laevis; Dm, Drosophila melanogaster; At, Arabidopsis thaliana*) of the WTAP-interacting region in VIRMA. The up-triangles indicate the residues that interact with WTAP-a (orange), WTAP-b (yellow), and ZC3H13 (orange red).
