# Supplementary material for: Cryo-EM structures of human m6A writer complexes
Source: Cell Res. 2022 Sep 27;32(11):982–94. doi: 10.1038/s41422-022-00725-8 (PMC9652331; doi:10.1038/s41422-022-00725-8)
Supplement: Supplementary file 6 — Supplementary information, Figure S6 [file 41422_2022_725_MOESM6_ESM.pdf]

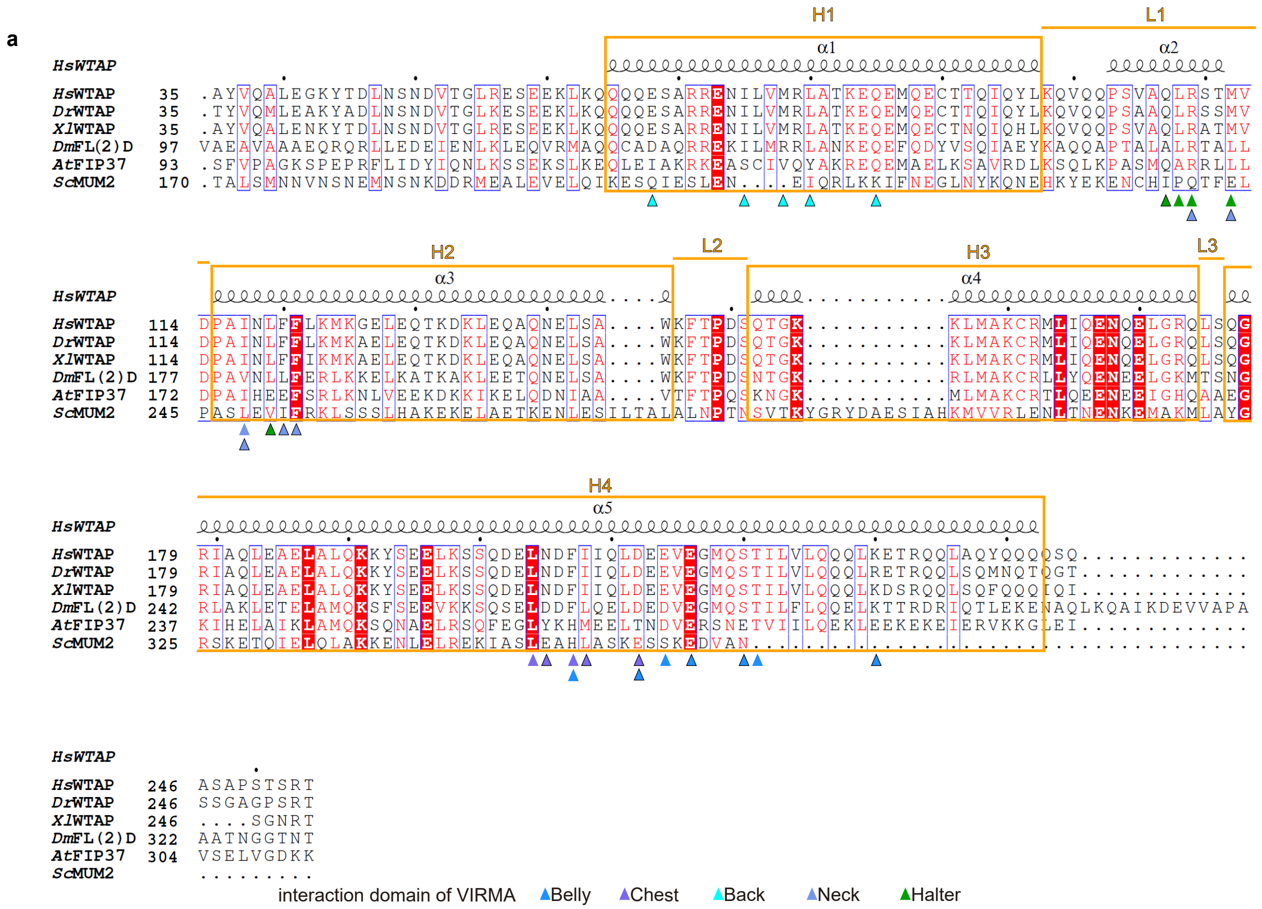

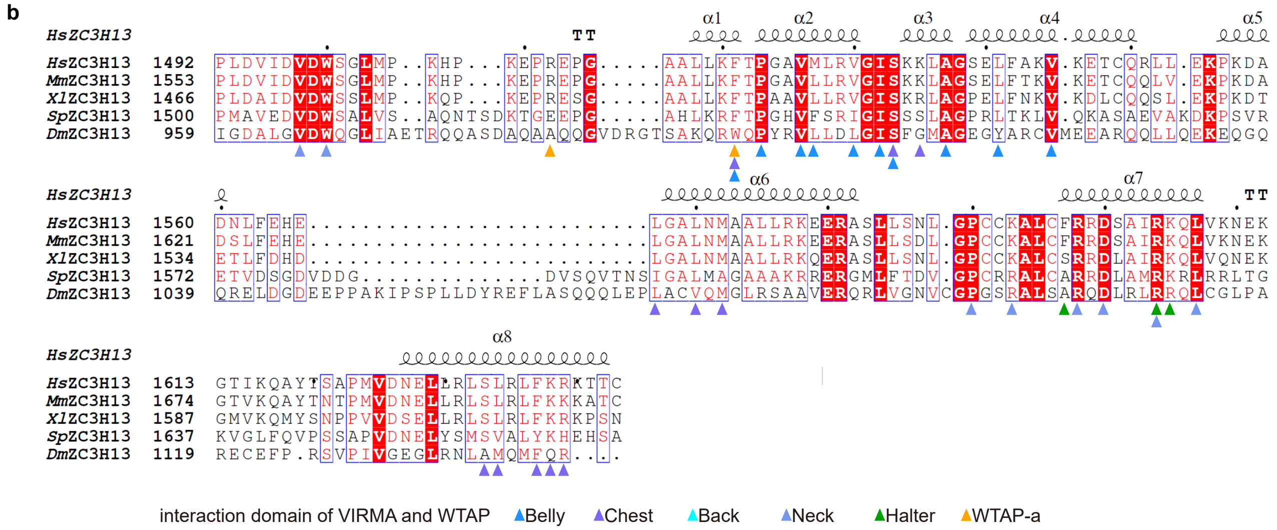

Supplementary information, Fig. S6. Comparison of homologous sequences of the WTAP H1-H4 and ZC3H13 Barding domains. a Alignment of sequences (*Hs, Homo sapiens; Dr, Danio rerio; Xl, Xenopus laevis; Dm, Drosophila melanogaster; At, Arabidopsis thaliana*) of WTAP H1-H4. The up-triangles with (WTAP-a) or without black-border (WTAP-b) indicate the residues that interact with VIRMA, colored by the corresponding domain of VIRMA. **b** Alignment of sequences (*Hs, Homo sapiens; Dr, Danio rerio; Xl, Xenopus laevis; Dm, Drosophila melanogaster; At, Arabidopsis thaliana*) of the ZC3H13 Barding domain. The up-triangles indicate the residues that interact with VIRMA, colored by the corresponding domain of VIRMA.
