# Supplementary material for: Cryo-EM structures of human m6A writer complexes
Source: Cell Res. 2022 Sep 27;32(11):982–94. doi: 10.1038/s41422-022-00725-8 (PMC9652331; doi:10.1038/s41422-022-00725-8)
Supplement: Supplementary file 8 — Supplementary information, Figure S8 [file 41422_2022_725_MOESM8_ESM.pdf]

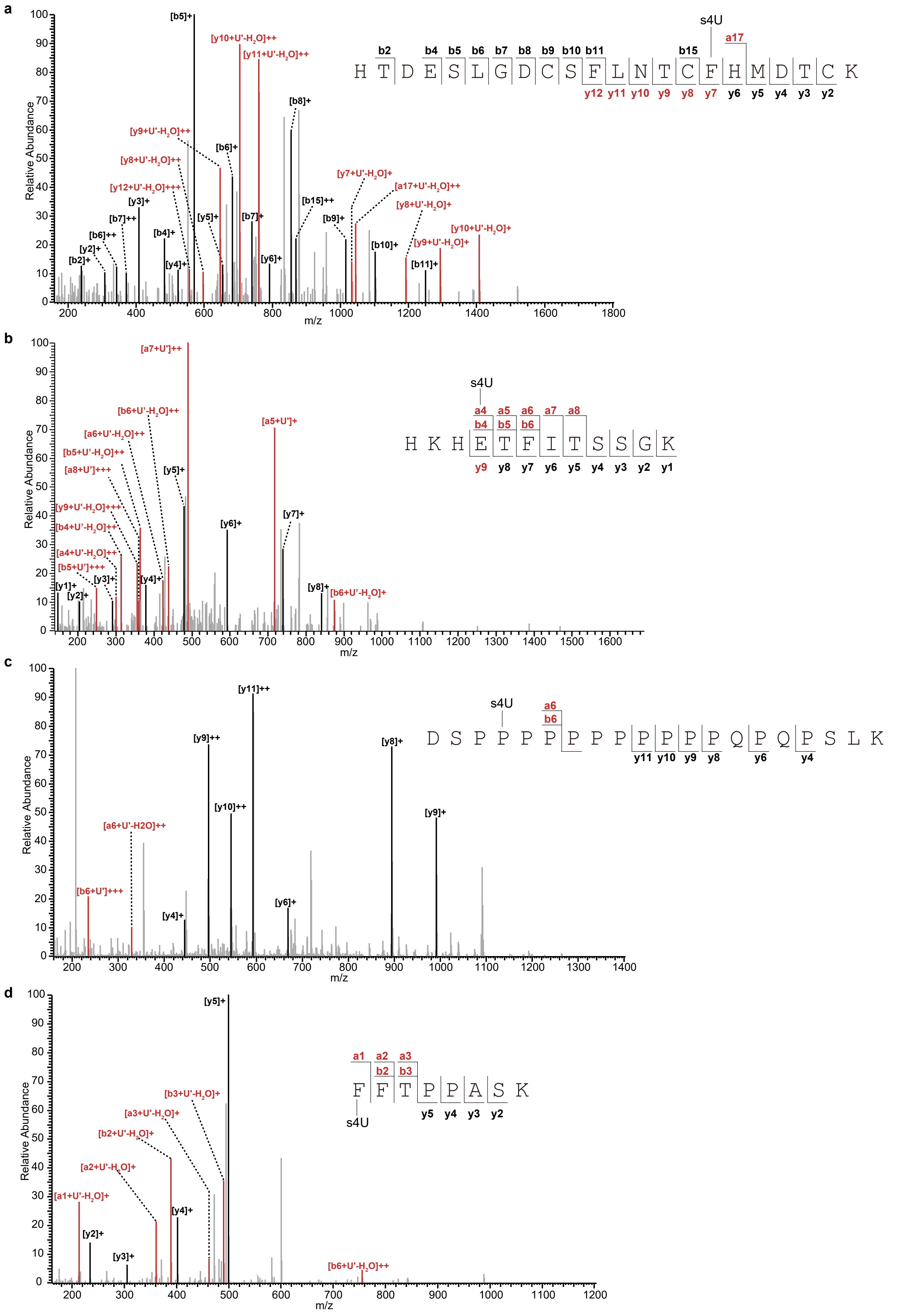

Supplementary information, Fig. S8. MS2 spectrum of a peptide-RNA mononucleotide (s4U minus H2S) crosslink derived from MAC/MACOM, corresponding to Fig. 7a. Raw data were search against a FASTA database consisting of the protein sequences of the proteins in the complexes using RNPxl node combined in PD software with 0.01 FDR.
