# Supplementary material for: Cryo-EM structures of human m6A writer complexes
Source: Cell Res. 2022 Sep 27;32(11):982–94. doi: 10.1038/s41422-022-00725-8 (PMC9652331; doi:10.1038/s41422-022-00725-8)
Supplement: Supplementary file 9 — Supplementary information, Figure S9 [file 41422_2022_725_MOESM9_ESM.pdf]

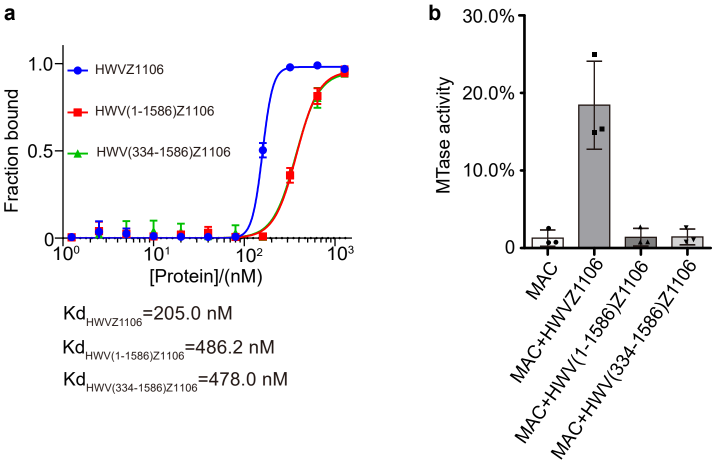

Supplementary information, Fig. S9. The C-terminal of VIRMA plays a vital role in the process of m^6A methylation. The N-terminal region (1-333) before the visible region and C-terminal region (1587-1812) after the Halter domain were truncated for RNA binding and methylation assay. **a** The binding affinities of $HWVZ^{(1106\text{-}1668)}$, $HWV^{(1\text{-}1586)}Z^{(1106\text{-}1668)}$, and $HWV^{(334\text{-}1586)}Z^{(1106\text{-}1668)}$ complex with FAM-labeled actb-1 RNA from EMSA experiments. Binding curves were generated using the mean of three biologically independent experiments, with standard deviation (SD) values indicated by error bars (n = 3). **b** Actb-1 RNA N6-adenosine methylation activity of MAC, $MAC/HWVZ^{(1106\text{-}1668)}$, $MAC/HWV^{(1\text{-}1586)}Z^{(1106\text{-}1668)}$, and $MAC/HWV^{(334\text{-}1586)}Z^{(1106\text{-}1668)}$ complex. The bar chart was generated using the mean of three biologically independent experiments, with standard deviation (SD) values indicated by error bars (n = 3).
