# Supplementary material for: Cryo-EM structures of human m6A writer complexes
Source: Cell Res. 2022 Sep 27;32(11):982–94. doi: 10.1038/s41422-022-00725-8 (PMC9652331; doi:10.1038/s41422-022-00725-8)
Supplement: Supplementary file 10 — Supplementary information, Figure S10 [file 41422_2022_725_MOESM10_ESM.pdf]

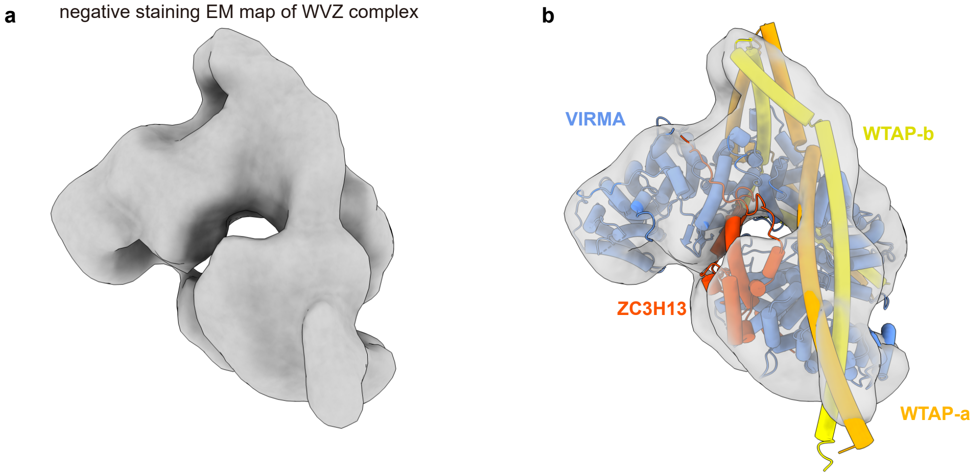

Supplementary information, Fig. S10. Structure of WVZ complex. a The negative staining EM map of WVZ complex. **b** Overall structure of the HWVZ complex fitted in the negative staining EM map of WVZ complex corresponding to **a**, with WTAP-a in orange, WTAP-b in yellow, ZC3H13 in orange-red, VIRMA in cornflower-blue, and the map of WVZ complex in grey translucent surface.
