# Supplementary material for: Cryo-EM structures of human m6A writer complexes
Source: Cell Res. 2022 Sep 27;32(11):982–94. doi: 10.1038/s41422-022-00725-8 (PMC9652331; doi:10.1038/s41422-022-00725-8)
Supplement: Supplementary file 11 — Supplementary information, Table S1 [file 41422_2022_725_MOESM11_ESM.pdf]

Table S1. Cryo-EM data collection, processing, and model validation

	HWVZ	HWV	HWVZ+M3/14
**Data collection and processing**			
Microscope	Titan Krios G3i	Titan Krios G3i	Titan Krios G3i
Voltage (kV)	300	300	300
Camera	Gatan K3	Gatan K3	Gatan K3
Grids Type	R2/1 Quantifoil copper	R2/1 Quantifoil copper	R2/1 Quantifoil copper
Sample concentration	1.0 mg/mL	1.0 mg/mL	1.0 mg/mL
Magnification	105,000x	105,000x	105,000x
C2 aperture size (μm)	70	70	70
Objective aperture size (μm)	100	100	100
Pixel size (Å)	0.82	0.82	0.82
Total exposure (e-/Å^2)	55.5	55.5	55.5
Exposure time (s)	2.5	2.5	2.5
Number of frames per exposure	30	30	30
Energy filter slit width (eV)	20	20	20
Data collection software	EPU	EPU	EPU
Number of exposures per hole	4	4	4
Defocus range (μm)	-1.1 to -3.3	-0.8 to -3.2	-1.0 to -3.5
Number of micrographs collected	12,745	7,642	14,168
Number of micrographs used	12,372	7,617	13,686
Number of initial particles	1,992,194	682,368	1,658,343
Symmetry	C1	C1	C1
Number of final particles	395,916	282,821	199,741

Clashscore, all atoms	9.76	8.31	N/A
Poor rotamers (%)	0.26	0	N/A
Favored rotamers (%)	95.86	99.64	N/A
Ramachandran outliers (%)	0	0	N/A
Ramachandran favored (%)	96.3	97.15	N/A
MolProbity score	1.76	1.60	N/A
Bad bonds (%)	0	0	N/A
Bad angles (%)	0.03	0.01	N/A
