# Supplementary material for: Cryo-EM structures of human m6A writer complexes
Source: Cell Res. 2022 Sep 27;32(11):982–94. doi: 10.1038/s41422-022-00725-8 (PMC9652331; doi:10.1038/s41422-022-00725-8)
Supplement: Supplementary file 12 — Supplementary information, Table S2 [file 41422_2022_725_MOESM12_ESM.pdf]

Table S2. Interface statistics generated by PDBsum structure bioinformatics analysis

	No. of interface residues	Interface area (Å^2)	No. of salt bridges	No. of hydrogen bonds	No. of non-bonded contacts
VIRMA vs ZC3H13	102:73	4209:4660	7	19	416
VIRMA vs WTAPa	57:44	2654:2902	3	15	250
VIRMA vs WTAPb	59:45	3055:3301	3	8	249
ZC3H13 vs WTAPa	6:5	258:253	1	3	32
WTAPa vs WTAPb	77:79	4637:4603	3	8	290
