# Supplementary material for: Cryo-EM structures of human m6A writer complexes
Source: Cell Res. 2022 Sep 27;32(11):982–94. doi: 10.1038/s41422-022-00725-8 (PMC9652331; doi:10.1038/s41422-022-00725-8)
Supplement: Supplementary file 13 — Supplementary information, Table S3 [file 41422_2022_725_MOESM13_ESM.pdf]

Table S3. Cross-linking sites of MAC and MACOM

Protein1(site)-Protein2(site)	#Spec-Total	Best E-value	Sample	Distance between Cα(Å)	Remark
WTAP(123)-WTAP(125)	20	4.18E-30	BS3-HWVZ	10.84	homodimeric link
WTAP(123)-WTAP(132)	4	1.30E-11	BS3-HWVZ	14.2	homodimeric link
WTAP(132)-WTAP(134)	19	4.15E-10	BS3-HWVZ	8.97	homodimeric link
WTAP(132)-WTAP(146)	28	4.61E-08	BS3-HWVZ	21.65	homodimeric link
WTAP(134)-WTAP(146)	37	2.68E-22	BS3-HWVZ	17.33	homodimeric link
WTAP(191)-WTAP(192)	10	3.83E-17	BS3-HWVZ	11.26	homodimeric link
WTAP(61)-WTAP(61)	5	2.52E-09	BS3-HWVZ		homodimeric link
WTAP(61)-WTAP(63)	52	5.26E-17	BS3-HWVZ		homodimeric link
WTAP(132)-WTAP(133)	4	4.28E-04	EDC-HWVZ	10.71	homodimeric link
WTAP(83)-WTAP(84)	3	7.24E-05	EDC-HWVZ	6.71	homodimeric link
HAKAI (195)-ZC3H13(1167)	7	6.01E-05	BS3-HWVZ+M3/M14		
HAKAI (195)-ZC3H13(1388)	4	1.81E-15	BS3-HWVZ+M3/M14		
HAKAI (195)-WTAP(63)	3	1.58E-08	BS3-HWVZ+M3/M14		
HAKAI (195)-WTAP(268)	3	8.32E-14	BS3-HWVZ+M3/M14		
HAKAI (195)-WTAP(192)	3	1.26E-07	BS3-HWVZ+M3/M14		
HAKAI (195)-WTAP(19)	10	2.44E-10	BS3-HWVZ+M3/M14		
HAKAI (195)-VIRMA(899)	3	1.02E-07	BS3-HWVZ+M3/M14		
HAKAI (195)-VIRMA(421)	3	4.21E-09	BS3-HWVZ+M3/M14		
HAKAI (195)-VIRMA(1713)	6	1.42E-08	BS3-HWVZ+M3/M14		
HAKAI (195)-METTL14(148)	3	1.69E-12	BS3-HWVZ+M3/M14		
METTL3(27)-VIRMA(457)	7	9.97E-07	BS3-HWVZ+M3/M14		*
METTL3(576)-VIRMA(899)	3	3.63E-04	BS3-HWVZ+M3/M14		*
METTL3(576)-VIRMA(1613)	3	5.75E-09	BS3-HWVZ+M3/M14		
METTL3(578)-VIRMA(1613)	3	3.35E-06	BS3-HWVZ+M3/M14		
METTL3(578)-VIRMA(1713)	3	2.99E-03	BS3-HWVZ+M3/M14		
METTL3(13)-WTAP(192)	14	1.28E-15	BS3-HWVZ+M3/M14		*
METTL3(27)-WTAP(230)	11	2.37E-29	BS3-HWVZ+M3/M14		*
METTL3(62)-WTAP(192)	5	5.54E-05	BS3-HWVZ+M3/M14		*
METTL3(132)-WTAP(192)	9	3.32E-34	BS3-HWVZ+M3/M14		*
METTL3(177)-WTAP(192)	11	7.52E-17	BS3-HWVZ+M3/M14		*
METTL3(177)-WTAP(155)	6	3.69E-09	BS3-HWVZ+M3/M14		*
METTL3(215)-WTAP(192)	5	8.18E-20	BS3-HWVZ+M3/M14		*
METTL3(215)-WTAP(160)	6	2.50E-06	BS3-HWVZ+M3/M14		*
METTL3(240)-WTAP(155)	3	3.47E-18	BS3-HWVZ+M3/M14		*
METTL3(241)-WTAP(160)	7	1.64E-07	BS3-HWVZ+M3/M14		*
METTL3(263)-WTAP(192)	3	7.83E-09	BS3-HWVZ+M3/M14		*
METTL3(263)-WTAP(160)	3	2.00E-07	BS3-HWVZ+M3/M14		*
METTL14(38)-VIRMA(399)	9	7.33E-19	BS3-HWVZ+M3/M14		*
METTL14(63)-VIRMA(880)	7	1.56E-16	BS3-HWVZ+M3/M14		*
METTL14(63)-VIRMA(899)	8	4.76E-23	BS3-HWVZ+M3/M14		*
METTL14(148)-VIRMA(880)	3	2.38E-19	BS3-HWVZ+M3/M14		*
METTL14(148)-VIRMA(887)	4	3.28E-12	BS3-HWVZ+M3/M14		*
METTL14(148)-VIRMA(1613)	4	6.64E-12	BS3-HWVZ+M3/M14		
METTL14(148)-VIRMA(899)	4	2.29E-08	BS3-HWVZ+M3/M14		*
METTL14(63)-WTAP(192)	9	1.59E-13	BS3-HWVZ+M3/M14		*
METTL14(148)-WTAP(192)	7	4.46E-19	BS3-HWVZ+M3/M14		*
METTL14(148)-ZC3H13(1255)	5	8.22E-14	BS3-HWVZ+M3/M14		
METTL14(148)-HAKAI(195)	3	1.69E-12	BS3-HWVZ+M3/M14		
METTL3(130)-ZC3H13(1182)	3	6.11E-09	EDC-HWVZ+M3/M14		*
METTL3(130)-ZC3H13(1509)	5	1.97E-11	EDC-HWVZ+M3/M14		*
METTL3(132)-ZC3H13(1554)	3	6.46E-07	EDC-HWVZ+M3/M14		*
METTL3(289)-WTAP(155)	7	6.00E-22	EDC-HWVZ+M3/M14		*
METTL3(256)-WTAP(170)	4	2.43E-05	EDC-HWVZ+M3/M14		*
METTL3(235)-WTAP(170)	3	4.19E-04	EDC-HWVZ+M3/M14		*
METTL3(130)-WTAP(192)	4	3.51E-22	EDC-HWVZ+M3/M14		*
METTL3(132)-WTAP(170)	5	3.25E-22	EDC-HWVZ+M3/M14		*
METTL3(139)-WTAP(192)	3	1.67E-08	EDC-HWVZ+M3/M14		*
METTL3(289)-VIRMA(899)	5	9.48E-10	EDC-HWVZ+M3/M14		*

*the visible MAC-cross-linked site in the HWVZ structure
