# Supplementary material for: Cryo-EM structures of human m6A writer complexes
Source: Cell Res. 2022 Sep 27;32(11):982–94. doi: 10.1038/s41422-022-00725-8 (PMC9652331; doi:10.1038/s41422-022-00725-8)
Supplement: Supplementary file 16 — Supplementary information, Video legend [file 41422_2022_725_MOESM16_ESM.pdf]

Supplementary information, Video S1: Overview of the cryo-EM structures of HWV and HWVZ complexes and the conformational changes upon ZC3H13 binding. The color scheme is used as in Fig. 2.

Supplementary information, Video S2: Model of the HWVZ+M3/M14 complex based on the cryo-EM map and biochemical results. The XL-MS cross-linking sites and METTL3/METTL14 are colored as in Fig. 6k.
